# Single-cell spatial transcriptomics reveals sex-dependent gene expression and intercellular signalling in mouse adrenal cortex

**DOI:** 10.1038/s42003-026-10697-9

**Published:** 2026-07-14

**Authors:** Małgorzata Blatkiewicz, Szymon Hryhorowicz, Marta Szyszka, Joanna Suszynska-Zajczyk, Andrzej Plawski, Anna Olechnowicz, Andrea Porzionato, Ludwik K. Malendowicz, Marcin Rucinski

**Affiliations:** 1 Spatial Transcriptomics Laboratory, Department of Histology and Embryology, Poznan University of Medical Sciences, Poznan, Poland; 2 Institute of Human Genetics, Polish Academy of Sciences, Spatial Multiomics Laboratory, Poznan, Poland; 3 Poznan University of Medical Sciences, Experimental Medicine Centre, Poznan, Poland; 4 Department of Biochemistry and Biotechnology, Poznan University of Life Sciences, Poznan, Poland; 5 Department of General and Endocrine Surgery and Gastroenterological Oncology, Poznan University of Medical Sciences, Poznan, Poland; 6 Department of Histology and Embryology, Poznan University of Medical Sciences, Poznan, Poland; 7 Department of Neuroscience, Institute of Human Anatomy, University of Padova, Padova, Italy; 8 Department of Bioinformatics and Computational Biology, Poznan University of Medical Sciences, Poznan, Poland

**Keywords:** Transcriptomics, Adrenal gland diseases

## Abstract

Sexual dimorphism profoundly influences adrenal physiology and disease susceptibility, yet its spatial molecular basis remains poorly understood. The female-predominant X-zone, a transient cortical structure recognized for nearly a century, has lacked comprehensive molecular characterization. Here we use Visium HD spatial transcriptomics with Cellpose-based cell segmentation to generate ~203,000 near-single-cell-resolution transcriptomic profiles from sexually mature mouse adrenal glands (four male, four female). We identify ten distinct cell populations with sex differences predominantly concentrated in inner cortical zones. The X-zone exhibits pronounced sexual dimorphism, with Akr1c18 as the primary marker (log₂FC = −16.28, female-enriched); combined use with Pik3c2g provides more robust zone identification, confirming the X-zone as a specialized progesterone-catabolizing compartment. Female adrenal glands exhibit greater intercellular communication complexity (435 vs 369 interactions) and higher aggregate signalling strength, with SPP1-integrin pathways predicted to be enriched in the female X-zone microenvironment. Spatial trajectory inference reveals a dominant centripetal gradient conserved across both sexes and a secondary centrifugal axis from the JMZ/X region prominent in females. This atlas establishes spatial restriction as a fundamental organizing principle of endocrine sexual dimorphism, with implications for precision medicine approaches to adrenal disorders.

## Abstract (web summary)

High-resolution Visium HD spatial transcriptomics of mouse adrenal glands shows that sexual dimorphism is spatially restricted to the inner cortex and defines the female X-zone as a specialized progesterone-catabolising compartment.

## Introduction

Sexual dimorphism profoundly shapes adrenal physiology and disease susceptibility, yet the molecular basis of sex-specific differences at single-cell spatial resolution remains incompletely characterized. The adrenal gland is a vital endocrine organ that regulates stress responses, metabolism, electrolyte homeostasis, and blood pressure through the secretion of steroid hormones and catecholamines^1,2^. The mammalian adrenal cortex exhibits a characteristic concentric zonation pattern, with distinct layers producing specialized steroid hormones: the zona glomerulosa (ZG) synthesizes mineralocorticoids (aldosterone), the zona fasciculata (ZF) produces glucocorticoids (corticosterone in rodents, cortisol in humans), and in some species, the zona reticularis secretes adrenal androgens^3,4^. This spatial organization reflects zone-specific expression of steroidogenic enzymes and differential hormonal regulation^5,6^. Emerging evidence demonstrates that the adrenal cortex is a dynamic tissue undergoing continuous cellular renewal through centripetal migration from progenitor populations located in the adrenal capsule and subcapsular region^7–9^. Lineage tracing studies have identified stem/progenitor cells marked by Sonic hedgehog (Shh)^10^, Wnt4^11^, Gli1^12^, or Rspo3^13^ expression that give rise to differentiated steroidogenic cells. Understanding these spatial differentiation trajectories has important implications for adrenal development, tissue homeostasis, and the pathogenesis of adrenocortical disorders including hyperplasia, adenomas, and carcinomas^14,15^.

Sexual dimorphism in adrenal structure and function has been recognized for nearly a century, yet the spatial molecular basis of these differences has not been comprehensively characterized at single-cell resolution^16–18^.The most striking sex difference in mice is the presence of X-zone, a transient cortical layer located between the zona fasciculata and medulla which is prominent in virgin females but largely absent in males^19,20^. This mouse-specific structure^21^, a remnant of the fetal adrenal cortex, undergoes involution after first pregnancy in females or during male puberty under androgenic influence^22,23^. Despite its marked sexual dimorphism and long-standing recognition, the molecular identity and functional significance of the X-zone remain elusive, as early studies relied primarily on histological criteria and limited biochemical characterization^24,25^. Over the past decade, significant progress has been made in deciphering the molecular mechanisms underlying adrenal sexual dimorphism. The androgen receptor (AR) and its transcriptional target *Hhex* have emerged as central regulators: androgens globally suppress gene transcription in zona fasciculata cells through NR5A1-mediated mechanisms^26^, and *Hhex* has been shown to maintain glucocorticoid levels and protect the adrenal cortex from androgen-induced lipid depletion^27^. Wnt signaling, senescence pathways, and sex-specific immune cell composition have also been implicated^28^. Transcriptomic profiling across postnatal development has revealed that sex-dependent differences in gene expression increase progressively, with male and female trajectories diverging markedly from postnatal day 28 onwards^29^. Furthermore, Grabek et al. demonstrated that female adrenal glands employ an additional stem and/or progenitor compartment, suggesting sex-specific mechanisms of cortical renewal^9^. Despite this progress, the spatial organisation of sex-biased gene expression and intercellular communication at near-single-cell resolution has not been comprehensively characterized.

Beyond anatomical differences, sex differences extend to HPA axis activity and stress responsiveness, with females generally displaying higher basal glucocorticoid levels and greater stress reactivity^30–33^. Many adrenal disorders exhibit sex biases, including Cushing’s syndrome and primary aldosteronism^34–38^, yet comprehensive molecular characterization of sex differences in adrenal tissue organization has been lacking.

Recent advances in spatial transcriptomics enable transcriptome-wide profiling while preserving tissue architecture^39,40^. High-resolution platforms such as Visium HD, combined with deep learning-based cell segmentation (Cellpose), now achieve near-single-cell resolution^41^. To date, the spatial organisation of sex-biased gene expression, the molecular identity of the X-zone at near-single-cell resolution, and the architecture of sex-dependent intercellular communication networks in the adrenal cortex have not been comprehensively characterized. Here we address these gaps and provide an interactive web resource enabling open exploration of the resulting spatial atlas (https://adrenal-spatall-transcriptomic.shinyapps.io/adrenalsex/).

The adrenal cortex is an integrated tissue in which steroidogenic, vascular, immune, and stromal cells coordinate hormone production through paracrine signaling^18,42–45^. Recent computational approaches enable systematic inference of intercellular signaling networks by quantifying coordinated expression of ligand-receptor pairs in spatially resolved datasets, providing unprecedented insights into tissue-level organization^46,47^. Whether male and female adrenal glands employ fundamentally different intercellular signaling architectures to coordinate endocrine function remains an open question with important implications for understanding sex-specific stress responses and disease susceptibility.

Here, we performed high-resolution Visium HD spatial transcriptomics combined with computational cell segmentation to achieve near-single-cell resolution profiling of adrenal glands from sexually mature male and female mice. We constructed a comprehensive cellular atlas, systematically characterized sex-biased gene expression and cellular organization, and mapped intercellular communication networks and their sexual dimorphism using advanced computational approaches. Our findings reveal that sexual dimorphism in the mouse shows spatial restriction to inner cortical zones, with distinct cellular composition, gene expression programs, and signaling architectures. The X-zone emerges as a molecularly distinct, female-enriched endocrine structure specialized for progesterone catabolism. These results provide a molecular framework for understanding sex-specific adrenal physiology and offer a foundational resource for investigating adrenal disorders in a sex-aware manner.

## Results

### Spatial transcriptomic atlas reveals sex-specific cellular organization of the mouse adrenal gland

**Fig. 1:**
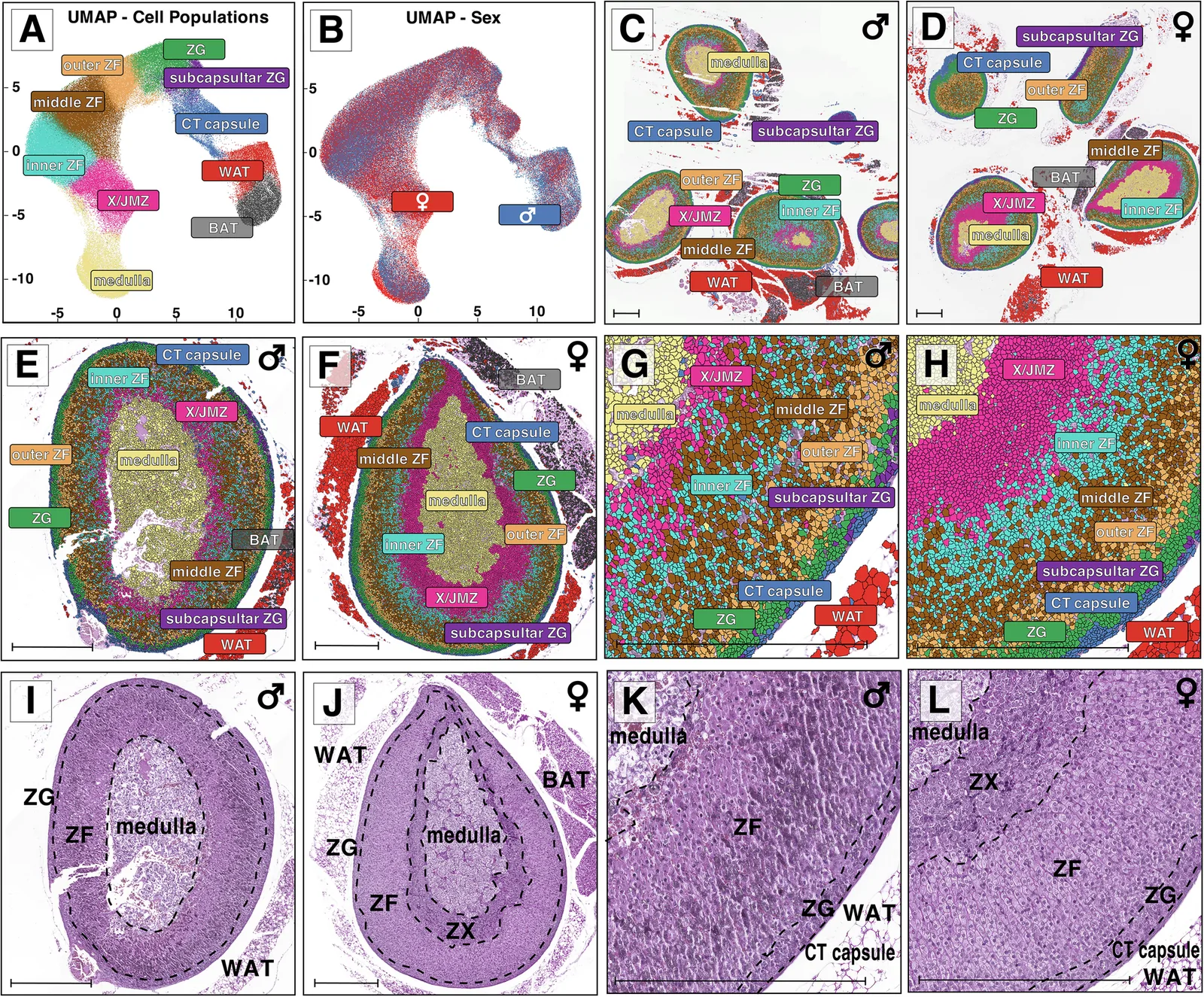
High-resolution spatial transcriptomics of male and female mouse adrenal glands reveals sex-specific cellular organization. **(A)** UMAP visualization of 203,069 Cellpose-segmented cells from four male and four female adrenal glands showing ten distinct cell populations: CT capsule, subcapsular ZG, ZG, outer ZF, middle ZF, inner ZF, X/JMZ, medulla, WAT, and BAT. (**B)** UMAP colored by sex showing integrated male (blue, *n* = 104,843) and female (red, *n* = 98,226) cells after Harmony batch correction. Spatial distribution of cell populations overlaid on H&E-stained tissue sections from males (**C**) and females (**D**). High-magnification spatial views of a single adrenal section illustrating detailed cortical zonation in males (**E**) and females (**F**). High-magnification spatial maps highlighting individual cell cluster distribution across cortical zones in males (**G**) and females (**H**). Representative H&E-stained sections from male (**I**, **K**) and female (**J**, **L**) adrenal glands at low (**I**, **J**) and high (**K**, **L**) magnification. Scale bars - 500 µm. *n* = 4 biologically independent animals per sex.

To comprehensively characterize the cellular composition and spatial organization of the mouse adrenal gland, we performed high-resolution spatial transcriptomics on adrenal tissues from from 7-week-old BALB/c mice (*n* = 4 males, 4 females). We obtained ~203,000 separated cell-level transcriptomes derived from Cellpose3-based segmentation of Visium HD data, following unsupervised clustering and UMAP dimensionality reduction, resolved into ten distinct cell populations (Fig. 1A), which we annotated based on established marker gene expression and spatial localization patterns: (1) capsular connective tissue (CT capsule), (2) subcapsular ZG (subcapsular zona glomerulosa), (3) ZG, (4) outer ZF (outer zona fasciculata), (5) middle ZF (middle zona fasciculata) (6) inner ZF (inner zona fasciculata), (7) X/JMZ (X-zone/juxtamedullary zone), (8) medulla, (9) white adipose tissue (WAT), and (10) brown adipose tissue (BAT). Spatial mapping of these cell populations onto tissue sections revealed the characteristic concentric zonation of the adrenal cortex, progressing from the outer fibroblast-rich capsule and CT capsule through the steroidogenic zones (subcapsular ZG → ZG → outer ZF → middle ZF → inner ZF) to the central catecholamine-producing medulla (Fig. 1C–F). Integration of male and female datasets using Harmony resulted in overlapping distributions in UMAP space (Fig. 1B), indicating successful batch correction and harmonization while preserving biological variation between sexes (male *n* = 104,843; female *n* = 98,226). The X/JMZ, a cortical layer positioned between the inner ZF and medulla, showed striking sexual dimorphism, comprising 20% of female cortex versus 9% in males, representing the most pronounced anatomical sex difference. This female-predominant region corresponds to the transient X-zone, while males display a less prominent juxtamedullary zone. High-magnification views (Fig. 1E–H) revealed detailed spatial relationships between cortical zones and demonstrated sharp boundaries between functionally distinct layers. Representative H&E-stained sections confirmed the anatomical zonation pattern, with the X-zone (ZX) clearly visible in females but absent in males (Fig. 1I–L).

### Cell type-specific marker genes define distinct adrenal populations with spatially restricted expression patterns in both males and females

**Fig. 2:**
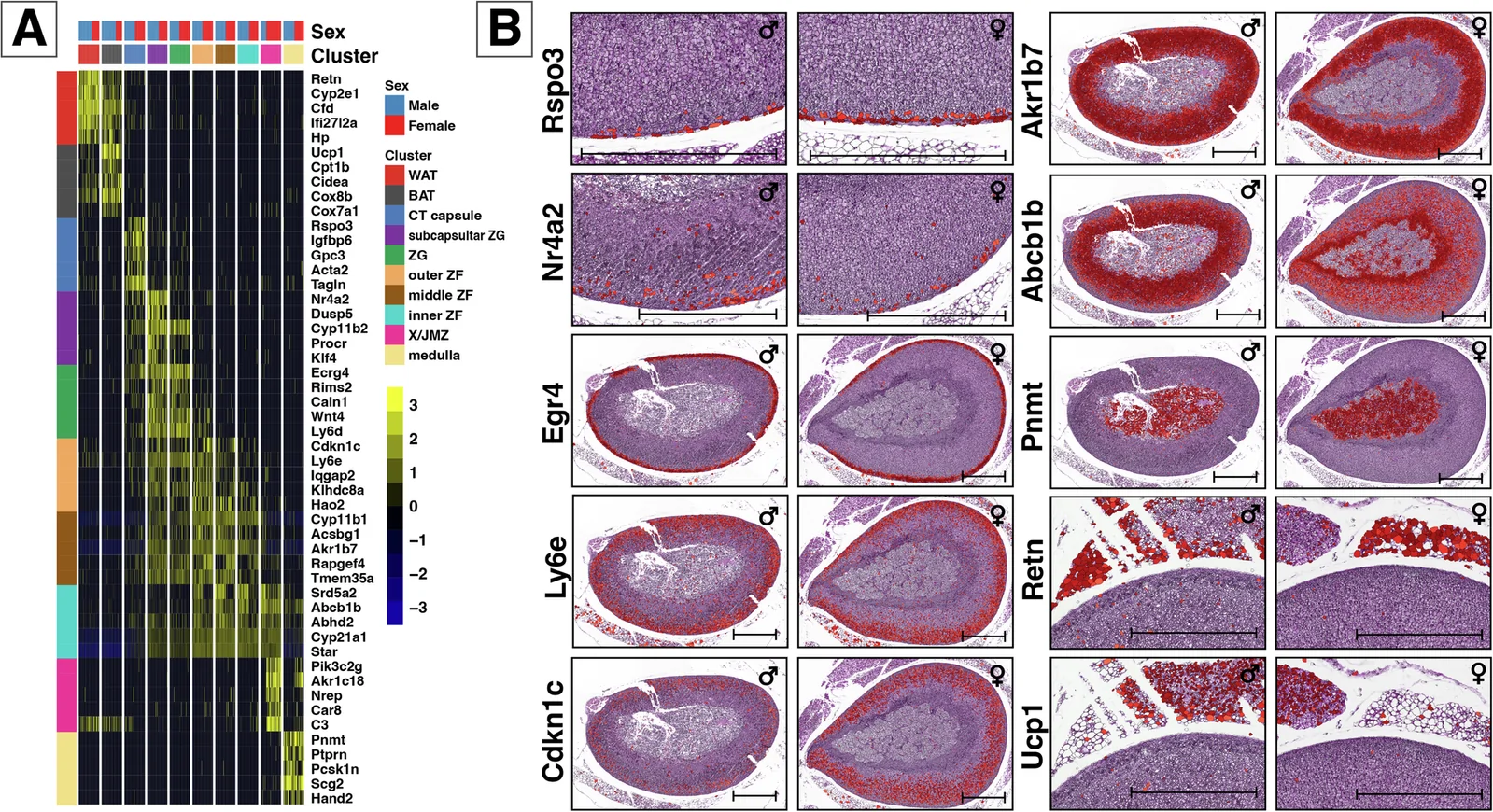
Cell type-specific marker gene signatures define adrenal cell populations with spatial validation in male and female mice. **(A)** Heatmap showing top marker genes for each adrenal cell population. Rows represent individual marker genes ordered by cell type, and columns represent individual cells (*n* = 100 per population) ordered by cell population (CT capsule, subcapsular ZG, ZG, outer ZF, middle ZF, inner ZF, X/JMZ, medulla, WAT, BAT) and by sex. Color intensity represents scaled expression level (z-score), with yellow indicating high expression and blue indicating low expression. (**B)** Spatial validation of selected marker genes across male (♂) and female (♀) adrenal tissue sections. Representative genes shown include: *Rspo3* (CT capsule), *Nr4a2* (subcapsular ZG), *Ecrg4* (ZG), *Ly6e* (outer ZF), *Ckdn1c* (middle ZF), *Akr1b7* (inner ZF), *Abcb1b* (X/JMZ), *Pnmt* (medulla), *Retn* (WAT), *Ucp1* (BAT). Images display expression patterns with high expression in red overlaid on H&E-stained tissue morphology. Presented spatial transcriptomic data are publicly accessible at https://adrenal-spatall-transcriptomic.shinyapps.io/adrenalsex/. Scale bars = 500 µm.

To molecularly characterize the ten identified cell populations, we performed differential expression analysis using the Wilcoxon rank-sum test on all integrated spots from both male and female samples. This analysis identified 2758 significantly differentially expressed marker genes across all populations (min.pct = 0.25, logFC.threshold = 0.25, adjusted *p* < 0.05), with each cluster exhibiting a distinct molecular signature (Supplementary Table 1). We selected the top 5 unique marker genes per population based on log2 fold-change. The resulting genes demonstrated clear population-specific expression patterns in a heatmap of 1000 downsampled spots (100 per population, ordered by sex) (Fig. 2A). Z-score normalized expression values revealed strong enrichment of each marker set within its cognate population in both males and females, with minimal cross-population expression, validating the distinctness of our cell type assignments and their conservation across sexes. Key marker genes for each population included Wnt signaling ligand for CT capsule (*Rspo3*), nuclear receptor and aldosterone synthase for subcapsular ZG (*Nr4a2*, *Cyp11b2*), signaling molecules for ZG (*Ecrg4*, *Wnt4*), cell cycle regulator for outer ZF (*Cdkn1c*), steroidogenic enzymes for middle ZF (*Cyp11b1* [11β-hydroxylase], *Akr1b7*), 5α-reductase for inner ZF (*Srd5a2*), aldo-keto reductases and phosphoinositide kinase for X/JMZ (*Akr1c18*, *Pik3c2g*), catecholamine synthesis machinery for medulla (*Pnmt* [phenylethanolamine N-methyltransferase] for epinephrine production), adipokines for WAT (*Retn* [resistin]), and thermogenic genes for BAT (*Ucp1* [uncoupling protein 1] for uncoupled respiration). Spatial mapping of representative marker genes in both male and female tissue sections confirmed their anatomically restricted expression patterns, while also revealing striking sex-specific differences (Fig. 2B). In both sexes, capsular markers (*Rspo3*) localized to the outermost tissue layer, subcapsular ZG and ZG markers (*Nr4a2*, *Ecrg4*) formed a distinct subcapsular band, outer ZF markers (*Ly6e*, *Cdkn1c*) occupied the mid-cortical region, inner ZF markers (Akr1b7) delineated the deep cortex, medullary markers (*Pnmt*) were confined to the central catecholaminergic core, and adipose markers (*Ucp1*) labeled peripheral fat depots. However, several markers displayed pronounced sex-specific spatial patterns. *Cdkn1c* showed stronger expression in females than males within the outer cortical zone. *Akr1b7* was expressed broadly throughout the cortex in males, whereas in females its expression was present throughout the cortex and in cells adjacent to the medulla, but was notably absent from the X-zone. Most strikingly, *Abcb1b* displayed pronounced sex-specific spatial distribution: in males, expression was detected broadly throughout the deep cortex, whereas in females overall cortical expression was reduced but markedly enriched within the X-zone.

### Sex-biased gene expression varies across adrenal cell populations

**Fig. 3:**
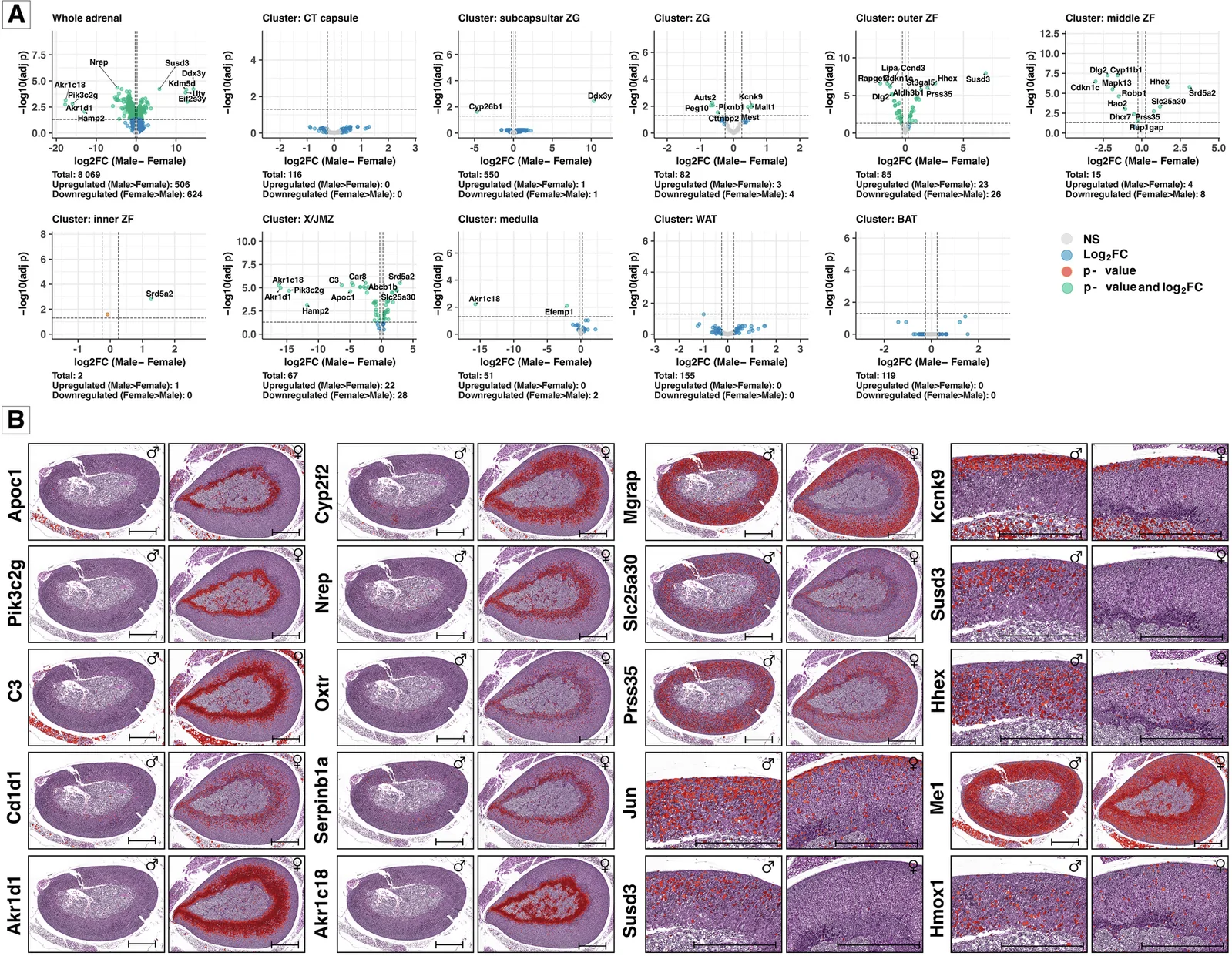
Sex-biased gene expression varies across adrenal cell populations. (**A**) Volcano plots showing differentially expressed genes between male and female mice at the whole-adrenal level and within individual cell populations. The x-axis represents log₂ fold-change (Male – Female). The y-axis represents -log₁₀(adjusted p-value). Genes meeting significance thresholds (adjusted *p* < 0.05 and |log₂FC | > 0.5) are colored in turquoise. Selected sex-biased genes are labeled. Numbers below each plot indicate total genes tested and significant upregulated (Male > Female) or downregulated (Female > Male) genes. (**B**) Spatial transcriptomic maps showing expression of representative sex-biased genes overlaid on H&E-stained tissue sections from male (♂) and female (♀) mice. The figure displays female-biased genes (*Apoc1, Pik3c2g, C3, Cd1d1, Akr1d1, Cyp2f2, Nrep, Oxtr, Serpinb1a, Akr1c18*) and male-biased genes (*Mgarp, Abcb1b, Prss35, Jun, Susd3, Konk9, Akr1b7, Hhex, Me1, Hmox1*) identified from differential expression analysis across adrenal cell populations. Color intensity represents normalized gene expression level. Presented spatial transcriptomic data are publicly accessible at https://adrenal-spatall-transcriptomic.shinyapps.io/adrenalsex/. Scale bars = 500 µm.

To systematically identify sex differences in gene expression, we performed pseudobulk differential expression analysis comparing male and female mice at both the entire adrenal level and within individual cell populations (Fig. 3). To ensure balanced and anatomically complete representation, pseudobulk analysis was performed using four male sections and two female sections that captured the full adrenal cross-section including the medullary compartment and X-zone; sections representing only peripheral cortical planes were excluded from this analysis. At the whole-adrenal level, we identified 1130 differentially expressed genes (adjusted *p* < 0.05, |log₂FC | > 0.5), with 506 genes showing male-biased expression and 624 genes showing female-biased expression (Fig. 3 first left top panel). Cluster-specific analysis revealed that sexual dimorphism was not uniformly distributed across cell populations, with most clusters showing minimal to modest sex-biased expression. The X/JMZ cluster exhibited the most pronounced sexual dimorphism of all adrenal cell populations, with 50 significantly differentially expressed genes (22 male-biased, 28 female-biased). Among female-biased genes, steroidogenic enzymes featured prominently. *Akr1c18* showed the most dramatic female enrichment (log₂FC = -16.28, adjusted *p* < 0.0001). Similarly, *Pik3c2g* (log₂FC = −14.65) and *Akr1d1* (log₂FC = −16.00) demonstrated extreme female bias. Male-biased genes in the X/JMZ included *Srd5a2* (5α-reductase 2; log₂FC = 2.96), Abcb1b (log₂FC = 2.33), and *Slc25a30* (log₂FC = 2.47). Complement pathway component *C3* demonstrated extreme female bias (log₂FC = −6.30). Apolipoprotein *Apoc1* (log₂FC = −4.93) showed pronounced female bias in the X/JMZ. The second most dimorphic cluster was outer ZF with 49 significantly differentially expressed genes (23 male-biased, 26 female-biased), followed by middle ZF with 15 differentially expressed genes (4 male-biased, 8 female-biased). Complete lists of differentially expressed genes with fold changes, adjusted p-values are provided in Supplementary Table 2.

To visualize the anatomical distribution of sex-biased gene expression across adrenal cell populations, we mapped the top 10 female-biased and top 10 male-biased genes onto H&E-stained adrenal tissue sections (Fig. 3B). Female-biased genes displayed striking spatial restriction to the inner cortical X-zone in female adrenals. *Apoc1*, *Pik3c2g*, *C3*, and *Cd1d1* showed intense, spatially restricted expression confined to the X-zone at the corticomedullary boundary. *Akr1c18* displayed the most sharply demarcated pattern, with dense expression forming a narrow band immediately adjacent to the medulla, including scattered cortical cells embedded within the medullary compartment. In contrast, *Akr1d1* and *Cyp2f2* exhibited broader, more diffuse expression extending throughout the inner cortical layers rather than being strictly confined to the X-zone boundary. This pattern of scattered *Akr1c18*-positive cells within the medullary compartment likely reflects the well-documented presence of steroidogenic cortical cells interdigitating within the chromaffin cell mass, identifiable by *Nr5a1* (Sf1) expression. We did not include *Nr5a1* immunostaining in the current validation panel; future studies should confirm the steroidogenic identity of these medullary *Akr1c18*-positive cells using Nr5a1 as a lineage marker.

*Mgarp*, *Prss35*, and *Susd3*, the latter encoding a complement regulatory protein implicated in cell adhesion and immune modulation, showed extensive expression throughout the cortical zones in males, while in females their expression was markedly reduced or spatially restricted. Ddx3y, a Y-chromosome-linked RNA helicase involved in transcriptional regulation and previously identified as a consistent male-specific adrenal marker^29^, was also among the top male-biased genes in the zona glomerulosa and outer zona fasciculata*. Hhex* and *Hmox1* displayed diffuse cortical expression in males, whereas female adrenals showed only scattered expression. *Jun* exhibited expression throughout the adrenal cortex in males; in females this gene showed predominantly subcapsular localization with weak cortical expression. *Abcb1b* showed broad deep cortical expression in males, while in females expression was reduced throughout the cortex but markedly enriched within the X-zone at the corticomedullary boundary. Similarly, *Akr1b7* showed broad cortical expression in males, while in females expression was present throughout the cortex but notably absent in the X-zone.

### Sex-specific cell-cell communication networks in the mouse adrenal gland

**Fig. 4:**
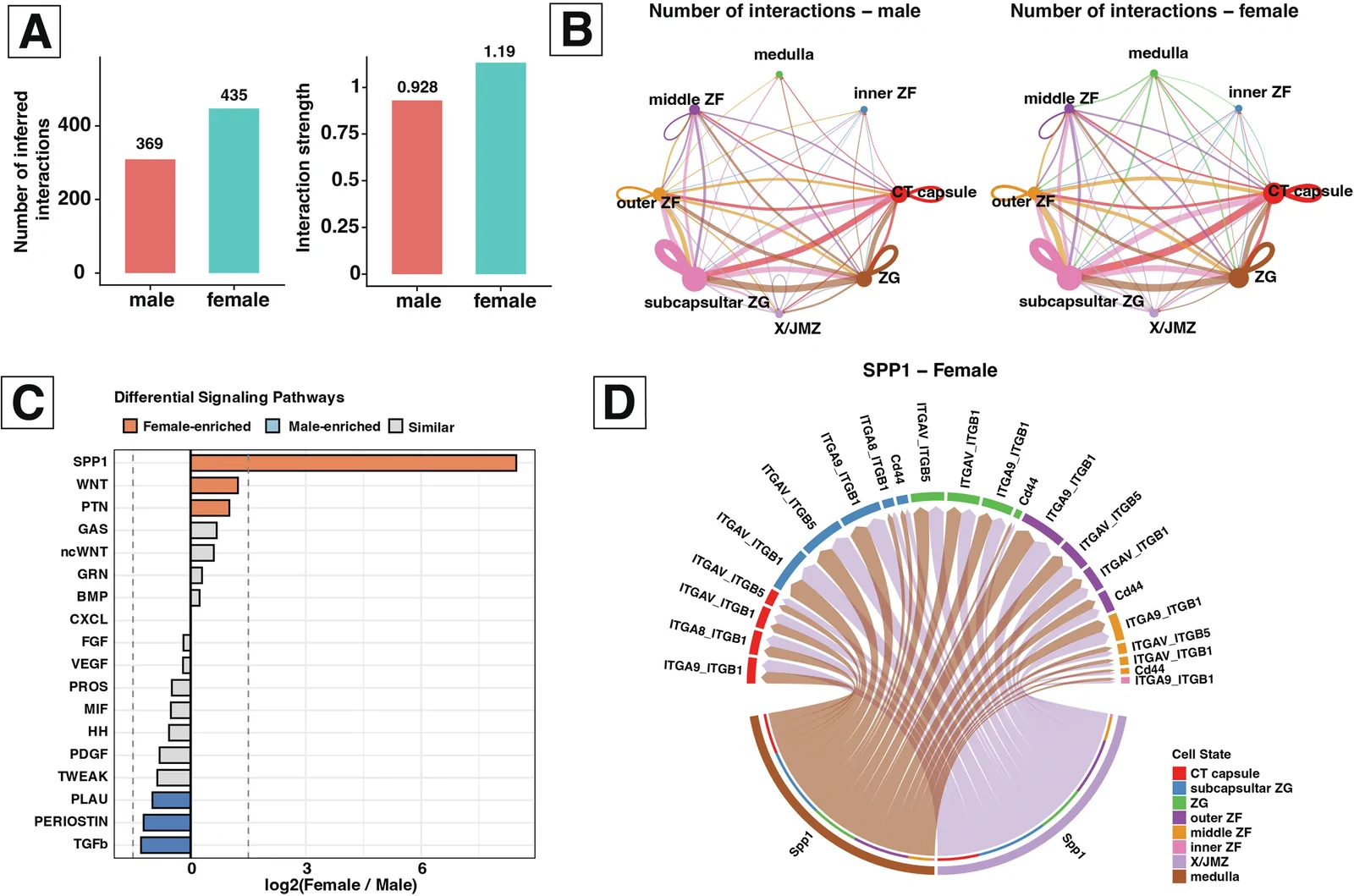
Sex-specific cell-cell communication networks in the mouse adrenal gland. (**A**) Bar plots comparing total number of inferred interactions (left) and aggregate interaction strength (right) in male and female adrenal glands. (**B**) Circle plots showing the number of interactions between cell populations in males (left) and females (right). Line thickness represents interaction number between cell type pairs. (**C**) Bar plot showing differential signaling pathways ranked by log₂(Female/Male) ratio. (**D**) Chord diagrams showing Spp1 (Secreted Phosphoprotein 1) signaling in females, illustrating complex multi-directional communication patterns between cell types including interactions with multiple integrin receptors (ITGAV_ITGB1, ITGAV_ITGB3, ITGAV_ITGB5, ITGAV_ITGB6, ITGAV_ITGB8, CD44).

**Fig. 5:**
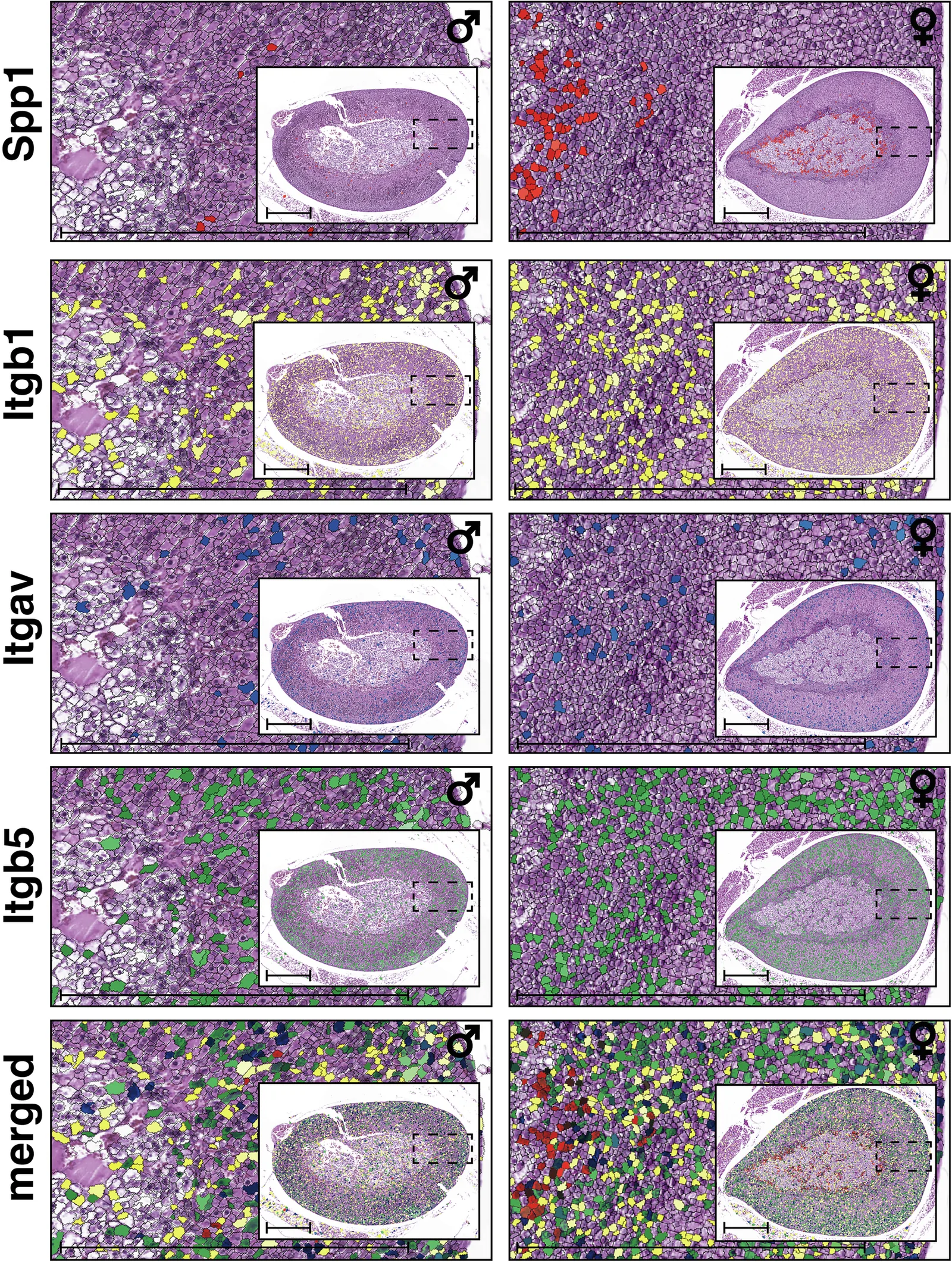
Spatial coexpression patterns of Spp1 signaling components in male (♂) and female (♀) mouse adrenal glands. (**A**) Spatial maps showing expression of *Spp1* ligand and its receptors *Itgav* (integrin subunit alpha V) *Itgb1* (integrin subunit beta 1) and *Itgb5* (integrin subunit beta 5) in individual spatial maps. Bottom merged panel shows co-localization of all four components. Color intensity represents normalized gene expression level. Scale bars = 500 µm.

To investigate whether sexual dimorphism extends beyond gene expression to intercellular signaling networks, we performed comprehensive cell-cell communication analysis using CellChat on four male and two female samples with complete adrenal cross-sections encompassing the medullary compartment and JMR/X-zone, after balanced downsampling to ensure fair statistical comparison (Fig. 4). To address potential biases from unequal cell numbers between sexes, all shared cell populations were downsampled to match the sex with fewer cells per cluster, resulting in fully balanced datasets. This approach equalized detection sensitivity while preserving spatial tissue architecture. Despite balanced cell numbers, global analysis revealed substantial differences in communication network complexity between sexes. Female adrenal glands exhibited 435 inferred interactions compared to 369 in males (Fig. 5A, left panel). Similarly, aggregate interaction strength was higher in females (1.19 vs 0.928) (Fig. 4A, right panel). Circle plot visualization confirmed this difference, showing a dense, highly interconnected network in females with extensive cross-talk among anatomical compartments, particularly between subcapsular ZG, ZG, and connective tissue capsule (Fig. 4B). Differential signaling pathway analysis revealed that SPP1, WNT, and PTN pathways were most strongly enriched in females, while TGFb, PERIOSTIN, and PLAU pathways showed male enrichment (Fig. 4C). SPP1 (Secreted Phosphoprotein 1, also known as Osteopontin) emerged as the most strongly female-enriched inferred signaling pathways. SPP1 signaling showed complex multi-directional communication patterns involving multiple integrin receptors (ITGAV_ITGB1, ITGAV_ITGB3, ITGAV_ITGB5, ITGAV_ITGB6, ITGAV_ITGB8) and CD44 (Fig. 4D). Spatial expression analysis indicated that inferred SPP1 signaling appears to originate predominantly from the medulla and X-zone.

To validate these computational predictions of sex-differential SPP1 signaling and visualize the spatial distribution of ligand-receptor components, we mapped *Spp1* and its integrin receptor subunits onto high-resolution Visium HD spatial sections (Fig. 5). *Spp1* expression was markedly enriched in the X-zone and medulla of female adrenals, with only sparse signal detected in males (Fig. 5, top row). The integrin receptor subunit *Itgb1* showed broad cortical expression in both sexes, while *Itgb5* was more widely distributed across cortical zones particularly in females. *Itgav* expression was low and scattered in both sexes. The merged composite images demonstrate extensive spatial overlap of ligand-receptor pairs in females, providing spatial co-expression support consistent with predicted SPP1-mediated intercellular communication in the female-specific adrenals.

### Spatial trajectory inference reveals transcriptional gradients consistent with centripetal zonation and a centrifugal axis emanating from the JMZ/X region

To investigate the spatial organization of cellular differentiation and migration within the adrenal cortex, we performed trajectory inference using SpaTrack, an optimal transport-based method that integrates transcriptomic similarity with spatial proximity to reconstruct developmental trajectories. Analyses were conducted separately for male (M1-M4) and female (F1–F4) samples to capture for potential sex-specific spatial patterns.

**Fig. 6:**
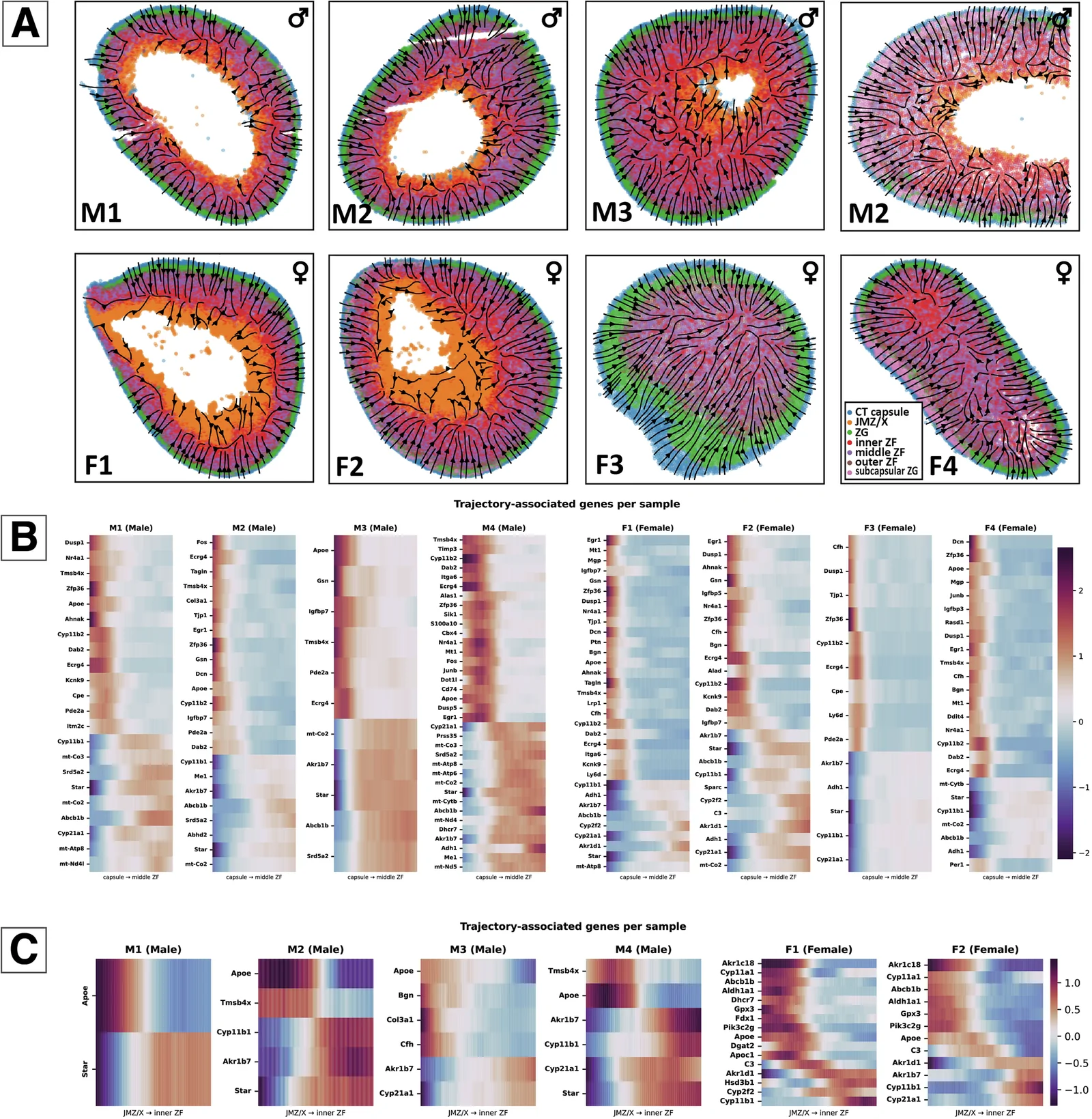
Spatial trajectory analysis. (**A**) Spatial velocity field analysis in male (M1–M4, top row) and female (F1–F4, bottom row) adrenal cortex. Cell populations colored by cluster identity. Black streamlines represent pseudotime progression estimated using optimal transport-based trajectory inference (SpaTrack). A dominant centripetal transcriptional gradient directed from the subcapsular zone toward the middle cortex is consistently observed across all samples and both sexes. (**B**) Heatmaps of trajectory-associated genes along the capsule-to-middle ZF pseudotime axis for all eight samples (M1–M4, F1–F4). Genes identified using Generalized Additive Models (model fit >0.15, FDR < 0.05). Rows represent genes ordered by peak expression along the trajectory. Color intensity indicates normalized expression (z-score). (**C**) Heatmaps of trajectory-associated genes along the JMZ/X-to-inner ZF axis in male samples (M1–M4) and female samples with complete corticomedullary representation (F1, F2).

Spatial velocity field analysis revealed a dominant centripetal transcriptional gradient in all eight samples, consistently directed from the CT capsule inward through the subcapsular ZG, ZG, and outer ZF zones toward the middle ZF (Fig. 6A). This capsule-to-cortex flow pattern was reproducible across biological replicates in both sexes. In addition, a secondary transcriptional gradient originating from the JMZ/X region adjacent to the medulla and extending outward toward the inner ZF boundary was detectable in samples with complete corticomedullary representation (Fig. 6A). This centrifugal axis was identified in all four male samples and in the two female samples (F1, F2) containing a well-defined X-zone; whereas the remaining female samples (F3, F4) lacked sufficient JMZ/X representation for reliable trajectory reconstruction. Notably, this centrifugal pattern was more prominent in female samples, consistent with the sexually dimorphic nature of the X-zone, although a comparable gradient was also detectable in males despite the smaller size of this region.

To identify genes dynamically regulated along these spatial trajectories, we performed Generalized Additive Model (GAM) analysis, testing for significant associations between gene expression and pseudotime. Pseudotime was anchored at the CT capsule (pseudotime = 0) and increased progressively toward the inner cortical regions. Genes were considered trajectory-associated if they showed strong model fit (R² > 0.15) and significant temporal dynamics (FDR < 0.05 after Benjamini-Hochberg correction).

Per-sample heatmaps revealed distinct temporal expression modules along the capsule-to-middle ZF axis across all eight samples (Fig. 6B). The both sexes, early trajectory genes expressed near the capsule included immediate-early response and transcription factors (*Egr1*, early growth response 1; *Fos*, FBJ osteosarcoma oncogene), extracellular matrix components (*Mgp*, matrix Gla protein; *Bgn*, biglycan; *Col3a1*, collagen type III alpha 1), and stress response regulators (*Dusp1*, dual-specificity phosphatase 1; *Ahnak*, neuroblast differentiation-associated protein), consistent with a progenitor-like transcriptional state. Mid-trajectory genes included steroidogenic enzymes (*Cyp21a1*, 21-hydroxylase; Star, steroidogenic acute regulatory protein; *Cyp11b1*, 11β-hydroxylase; *Cyp11b2*, aldosterone synthase) and the cortical potassium channel Kcnk9. Late trajectory genes, expressed toward the middle ZF, included lipid and steroid metabolism genes (*Apoe*, apolipoprotein E; *Akr1b7*, aldo-keto reductase 1B7), a cytoskeletal regulator (*Tmsb4x*, thymosin beta-4), and a multidrug efflux transporter (*Abcb1b*, P-glycoprotein). This progression from progenitor markers through steroidogenic enzymes toward lipid metabolism and transport genes mirrors the functional zonation of the adrenal cortex and supports a model of progressive transcriptional differentiation along the capsule-to-cortex axis. GAM analysis was additionally performed along the secondary centrifugal trajectory originating from the X/JMZ region toward the inner ZF boundary, in male samples (M1–M4) and female samples with complete corticomedullary representation (F1, F2). Trajectory-associated genes along this axis included established X-zone markers *Akr1c18* (20α-hydroxysteroid dehydrogenase), *Pik3c2g* (phosphatidylinositol-3-kinase), *Akr1d1* (5β-reductase), and *Spp1* (osteopontin), as well as additional inner cortex-enriched transcripts. The identification of these established X/JMZ markers among the top GAM-associated genes along this axis supports the biological validity of the inferred centrifugal trajectory and is consistent with a distinct transcriptional program emanating from the X/JMZ compartment, separate from the capsule-driven centripetal axis.

Cross-sex comparison of trajectory-associated genes revealed both conserved and sex-enriched expression patterns (Supplementary Fig. S2A). Genes consistently identified across ≥2 samples of each sex and ordered by peak pseudotime position in females showed that core steroidogenic genes (*Cyp21a1*, *Star*, *Akr1b7*, *Cyp11b1*) were active at comparable pseudotime positions in both sexes, indicating conservation of the fundamental steroidogenic differentiation program. Female-enriched early trajectory genes included the immediate-early gene *Fos*, cytoskeletal regulator *Gsn* (gelsolin), and ECM-associated growth modulator *Dcn* (decorin), as well as the stress response phosphatase *Dusp1*, while male samples showed relatively greater enrichment of extracellular matrix components at early pseudotime positions.

Analysis of the centrifugal JMZ/X-to-inner ZF trajectory identified trajectory-associated genes in all four male samples and in female samples F1 and F2 with complete corticomedullary representation (Fig. 6C). Female samples showed prominent expression of established X-zone markers (*Akr1c18*, aldo-keto reductase 1C18; *Akr1d1*, steroid 5β-reductase; *Cyp11a1*, cholesterol side-chain cleavage enzyme; *Aldh1a1*, aldehyde dehydrogenase 1A1) along this axis, consistent with the transcriptionally active X-zone in pre-pubertal females. In male samples, expression of these markers along the centrifugal axis was markedly reduced, reflecting the regressed or absent X-zone characteristic of the male adrenal gland. Cross-sex comparison of the centrifugal trajectory further highlighted these differences (Supplementary Fig. S2B). These transcriptional gradients along the JMZ/X-to-inner ZF axis are consistent with zone-specific gene expression patterns rather than obligatory directional cell migration, and should be interpreted as reflecting transcriptional states along a spatial axis rather than confirmed lineage trajectories in the absence of direct lineage tracing data.

### Immunohistochemical validation of sex-biased gene expression patterns in mouse adrenal glands

**Fig. 7:**
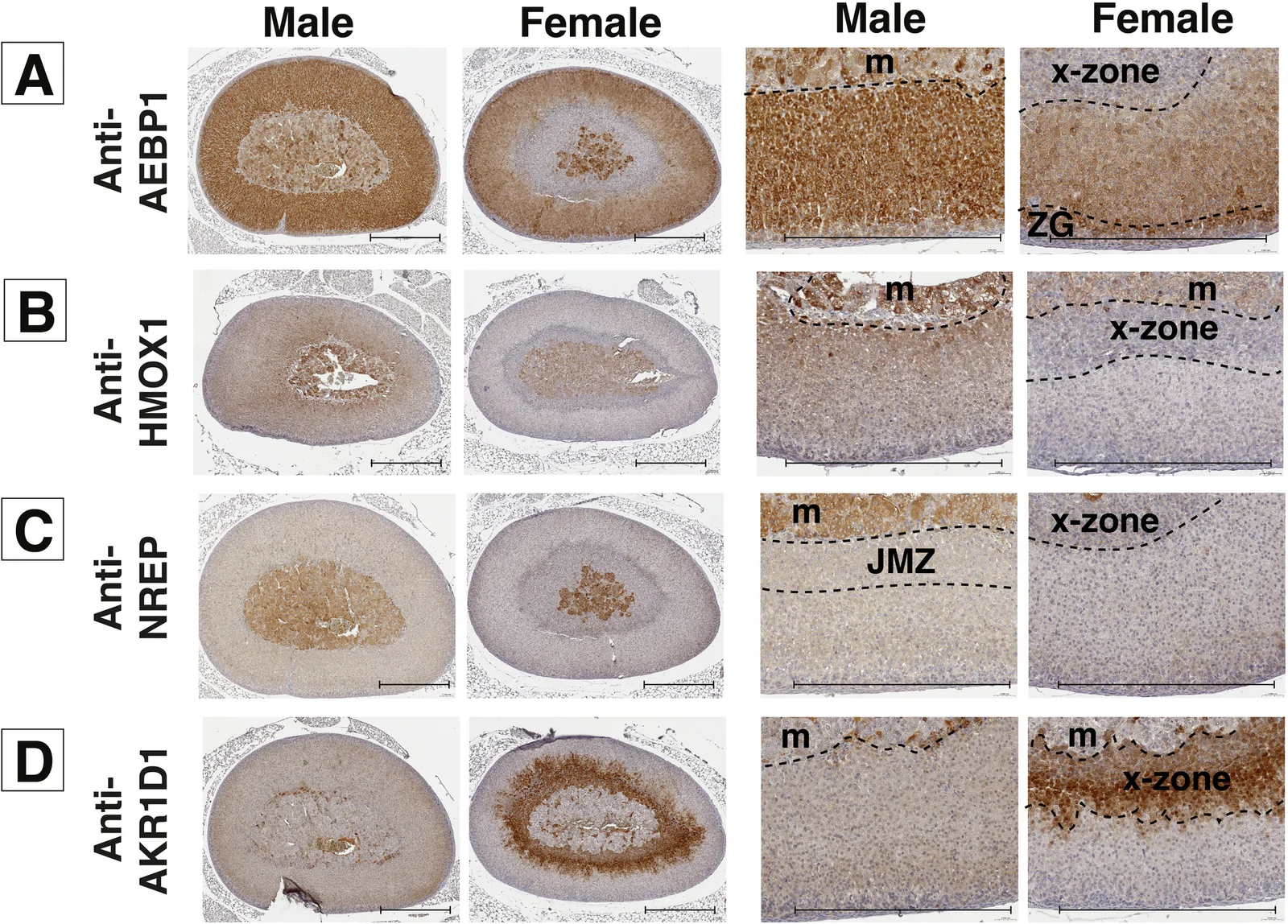
Immunohistochemical validation of sex-biased protein expression in the X/JMZ region. (**A**) Representative immunohistochemistry images showing expression of male-biased proteins AEBP1 and (**B**) HMOX1, and (**C**) NREP and (**D**) AKR1D1 in adrenal glands from male (left) and female (right) mice. First two panels illustrate low-magnification (100x) views of the entire adrenal sections, while the next two panels depict high-magnification (400x) images of the corticomedullary region. The presence of brown staining is an indication of positive immunoreactivity (DAB chromogen); sections are counterstained with hematoxylin.

To validate sex-biased gene expression patterns identified by spatial transcriptomics in the X/JMZ cluster (Fig. 4), we performed immunohistochemical staining for selected proteins in male and female adrenal glands (Fig. 7).

Male-biased proteins AEBP1 and HMOX1 showed robust cytoplasmic staining throughout the male adrenal cortex, with particularly intense expression in the juxtamedullary zone. AEBP1 (Aortic Carboxypeptidase-Like Protein) is a transcriptional repressor with established roles in adipogenesis, inflammation, and extracellular matrix remodeling; its male-biased adrenal expression suggests a potential role in sex-specific regulation of stromal or steroidogenic cell function. Female adrenals displayed markedly reduced immunoreactivity across all cortical zones, with near-absence in the X-zone, confirming male-specific expression patterns. HMOX1 exhibited the most dramatic sex difference, with strong cortical staining in males but virtual absence in the female X-zone. Of note, both AEBP1 and HMOX1 immunostaining produced detectable signals in the medulla of both male and female adrenal glands, a pattern not prominently reflected in the spatial transcriptomic data for either marker. This discrepancy is adressed below.

Female-biased proteins displayed complementary patterns. AKR1D1 demonstrated striking female-specific enrichment, with intense immunoreactivity concentrated at the corticomedullary boundary in females and only weak, scattered expression in males. Notably, the AKR1D1 protein distribution appeared broader than the sharply demarcated *Akr1c18* mRNA pattern, extending into the inner cortical layers beyond the strict X-zone boundary, consistent with the wider spatial expression of *Akr1d1* mRNA observed in the spatial transcriptomic maps. This partial mismatch between *Akr1d1* mRNA and AKR1D1 protein localization likely reflects post-transcriptional regulation or differences in protein stability, indicating that although *Akr1d1* is strongly female-enriched, its functional domain extends beyond the canonical X-zone marker *Akr1c18*. Strong cytoplasmic immunoreactivity was uniformly distributed throughout X-zone cells, validating the extreme female enrichment observed transcriptomically. NREP showed moderate female-biased expression with subcapsular and scattered X-zone localization, though spatial restriction was less pronounced than AKR1D1.

These findings confirm that the most pronounced sexual dimorphism at the protein level is concentrated at the corticomedullary boundary, where male-biased proteins (AEBP1, HMOX1) are virtually absent from the female X-zone and female-biased steroidogenic enzymes (AKR1D1, NREP) display strong X-zone enrichment. This is consistent with the broader transcriptomic picture, in which genes such as *Hhex*, *Mgarp*, *Jun*, and *Acsbg1* show male-biased expression extending across multiple cortical zones — reflecting that sexual dimorphism in the mouse adrenal cortex is most pronounced at the corticomedullary boundary but not exclusively confined to it. Coordinate regulation at mRNA and protein levels confirms the X-zone as a molecularly distinct, female-specific endocrine compartment.

## Discussion

This study presents the first comprehensive high-resolution spatial transcriptomic atlas of the sexually mature mouse adrenal gland at near-single-cell resolution, revealing profound sexual dimorphism that is predominantly concentrated in inner cortical zones. Notably, while the most extreme sex differences, including the X-zone molecular signature, are restricted to the corticomedullary boundary, selected male-biased genes such as *Hmox1* exhibit diffuse expression across multiple cortical zones, indicating that sexual dimorphism in the mouse adrenal cortex is zonal in emphasis rather than absolute in anatomical confinement. Using Visium HD spatial transcriptomics with Cellpose-based cell segmentation on samples from four male and four female 7-week-old mice, we demonstrate that the female-predominant X-zone represents a molecularly distinct steroidogenic compartment specialized for progesterone catabolism through 20α-hydroxysteroid dehydrogenase (*Akr1c18*) and is associated with predicted osteopontin (SPP1)-mediated signaling enriched in females. Female adrenal glands exhibited greater intercellular communication complexity (435 vs 369 interactions) and higher aggregate interaction strength (1.19 vs 0.928), while males engaged distinct pathway-specific mechanisms including TGFb, PERIOSTIN, and PLAU signaling. These findings establish spatial restriction as a fundamental organizing principle of endocrine sexual dimorphism and provide a molecular framework for understanding sex differences in adrenal disorders, with implications for precision medicine approaches.

Our spatial transcriptomic analysis confirmed the well-established concentric zonation of the adrenal cortex, with molecularly distinct zones expressing characteristic steroidogenic enzymes: *Cyp11b2* (aldosterone synthase) in the ZG, *Cyp11b1* (11β-hydroxylase) in the ZF, and zone-specific aldo-keto reductases in the inner cortex and X-zone^48–51^. The identification of a distinct subcapsular ZG population enriched for Wnt signaling components (*Rspo3*) aligns with previous lineage tracing studies demonstrating that Wnt/β-catenin signaling maintains the capsular progenitor/stem cell niche responsible for centripetal cortical renewal^10,13,52–54^. Beyond the traditional tripartite zonation, our data provide spatial evidence for functional sub-zones within the zona fasciculata, a concept recently proposed by Lyu and colleagues based on RNA-seq and immunohistochemistry^55^. This illustrates that bulk-level transcriptomic approaches, including RNA-seq and microarray analysis, already held the potential to reveal novel zone-specific molecular signatures in the adrenal gland when applied to comparisons across physiological conditions such as sex or age, yet without spatial resolution, the precise anatomical boundaries of these molecular domains could not be established. Lyu et al. demonstrated that the mouse zona fasciculata can be subdivided into at least three concentric sub-zones based on partially overlapping expression of inner cortex markers including *Cyp2f2*, *Dhcr24*, *Spp1*, and *Akr1c18* (20α-hydroxysteroid dehydrogenase, 20α-HSD)^55^. Our high-resolution spatial maps confirm and extend these findings, revealing sharp spatial boundaries between *Cyp11b1*-high outer ZF, steroidogenic enzyme-expressing middle ZF (*Cyp11b1, Akr1b7*), and the metabolically distinct inner ZF characterized by enrichment of lipid metabolism genes (*Apoe*, *Selenop*) and mitochondrial respiratory chain components. This spatial transcriptional heterogeneity within the zona fasciculata likely reflects functional specialization related to differential ACTH sensitivity, vascular architecture, or metabolic zonation.

The X-zone exhibited extreme female specificity of *Akr1c18* expression (log₂FC = −16.28). However, given that *Akr1c18* expression has been reported in cells of the definitive lineage in certain mouse models (e.g. *Prkar1a* and *Hhex* knockouts), we suggest caution in applying it as a universal gold-standard marker, and propose that its use in combination with *Pik3c2g*, which shows consistent X-zone specificity across models, provides more robust zone identification. Beyond *Akr1c18*, we identified additional female-enriched X-zone markers including *Akr1d1* (5β-reductase), *Pik3c2g*(phosphatidylinositol-3-kinase), and *Cd1d1* (lipid antigen presentation molecule), suggesting multifaceted functions beyond simple progesterone catabolism. *Pik3c2g* in particular has been reported as an X-zone marker in previous studies and may regulate steroidogenic enzyme trafficking or lipid droplet dynamics^56^. The co-expression of multiple steroid-metabolizing enzymes (*Akr1c18*, *Akr1d1*, *Pik3c2g*) suggests that the X-zone possesses unique steroidogenic capacity for both inactivation (20α-reduction of progesterone) and potentially activation (5α-reduction of androgens) of steroid hormones, confirming its identity as a distinct endocrine compartment rather than merely a vestigial fetal structure.

One of the most notable inferred findings from our cell-cell communication analysis was the identification of SPP1 (Secreted Phosphoprotein 1, also known as osteopontin) as the most strongly female-enriched predicted signaling pathway. Inferred SPP1 signaling exhibited complex multi-directional communication patterns involving multiple integrin receptors and CD44, with predicted signaling originating predominantly from medullary cells adjacent to the cortex and X/JMZ region. Spatial validation confirmed that *Spp1* ligand and its receptors (*Itgav*, *Itgb1*, *Itgb5*) show extensive co-localization in the female X-zone and adjacent medulla, with minimal expression in males, providing spatial co-expression patterns consistent with predicted SPP-1 mediated intercellular communication in the female-specific X-zone microenvironment. This finding is consistent with and extends recent observations by Lyu et al., who identified SPP1 as an inner cortex marker gene in mouse adrenal glands and demonstrated by immunohistochemistry that SPP1 protein localizes to the inner cortex partially colocalizing with the X-zone marker 20α-HSD^55^. Importantly, Lyu et al. identified SPP1 as one of the top sexually dimorphic genes responsive to thyroid hormone (T3), suggesting hormonal regulation of SPP1 expression^55^. Our high-resolution spatial data are consistent with these observations and suggest that SPP1 signaling may be spatially enriched in the female X-zone microenvironment; however, we wish to be explicit that these observations are correlative and do not establish a functional role for SPP1 in X-zone maintenance. A recently published bulk RNA-seq study profiling the mouse adrenal gland transcriptome across sex and postnatal age (Lyu et al.) provides independent computational support for these observations. In this dataset, *Spp1* co-segregates with established inner cortex markers (*Akr1c18*, *Cyp2f2*, *Pik3c2g*, *Dhcr24*) within a WGCNA co-expression module showing pronounced sex- and age-dependent regulation, with *Spp1* ranking fourth by fold-change between postnatal day 21 and day 49 in males, a pattern fully consistent with our spatial transcriptomic findings.

Osteopontin is a multifunctional matricellular glycoprotein with established roles in cell adhesion, migration, survival, and extracellular matrix remodeling^57,58^. In other tissues, SPP1 has been shown to (i) promote cell survival and inhibit apoptosis through integrin-mediated signaling, (ii) regulate immune cell recruitment and activation, (iii) modulate vascular permeability and angiogenesis, and (iv) coordinate tissue remodeling during development and injury responses^59–64^. Whether SPP1-integrin signaling is required for X-zone survival, regulation of *Akr1c18* expression, or resistance to androgen-mediated involution cannot be determined from transcriptomic data alone. *Spp1* knockout mice are viable and fertile and would represent an ideal model to test whether loss of osteopontin signaling affects X-zone morphology, molecular identity, or involution dynamics. We therefore propose SPP1-integrin signaling as a hypothesis-generating observation and a priority target for future functional investigation.

Our spatial trajectory inference analysis using SpaTrack revealed a dominant centripetal transcriptional gradient in all eight samples, directed from the CT capsule inward through successive cortical zones (subcapsular ZG → ZG → outer ZF → middle ZF), consistent with decades of lineage tracing studies demonstrating continuous centripetal migration from capsular progenitor populations. In addition, a secondary transcriptional gradient originating from the JMZ/X region and directed outward toward the inner ZF boundary was detectable in samples with complete corticomedullary representation (all four male samples and two female samples, F1 and F2). This centrifugal axis was particularly prominent in females, where the expanded X-zone showed abundant velocity streamlines directed toward the cortical-X-zone interface, and was also detectable in males despite their smaller JMZ/X region.This bidirectional organization is consistent with classical lineage tracing studies demonstrating that the transient (X-zone) and definitive (ZG + ZF) cortices descend from distinct fetal progenitor populations^65^. Zubair et al. used Cre-loxP fate mapping to show that the fetal X-zone (fetal adrenal cortex) and the adult definitive cortex arise from separate progenitor compartments during development, with the X-zone representing a fetal-derived population that persists postnatally in females^65^. Our spatial velocity patterns provide transcriptomic evidence that these two lineage-distinct compartments maintain separate organizational dynamics in the adult adrenal, with the X-zone exhibiting autonomous spatial trajectories rather than simply representing the terminal differentiation state of capsule-derived cells. It has to be noted that spatial velocity vectors inferred from transcriptomic data do not necessarily imply physical cell migration but rather reflect coordinated changes in transcriptional state across tissue space. The observed bidirectional patterns could arise from (i) true bidirectional cell migration, (ii) metabolic or functional gradients established by paracrine signaling, or (iii) spatial patterns of gene expression unrelated to cell movement^66,67^. Definitive resolution of whether X-zone cells physically migrate toward the cortex or whether the observed patterns reflect metabolic zonation will require time-resolved spatial transcriptomics or in vivo lineage tracing with spatial barcoding technologies.

Nevertheless, the trajectory-associated gene expression patterns we identified provide valuable insights into spatial functional specialization. The progression from immediate-early response genes and extracellular matrix components (*Egr1*, *Fos*, *Mgp*, *Bgn*) near the capsule → steroidogenic enzymes (*Star*, *Cyp21a1*, *Cyp11b1*, *Cyp11b2*) in mid-cortex → lipid metabolism and transport genes (*Apoe*, *Akr1b7*, *Abcb1b*) toward the middle ZF mirrors the known functional zonation of the adrenal cortex and validates the biological relevance of the inferred trajectories.

Despite balanced downsampling ensuring equal cell numbers across all clusters between sexes, global analysis revealed that female adrenal glands exhibit more inferred interactions (435 vs 369) and higher aggregate signaling strength (1.19 vs 0.928) compared to males. This finding is consistent with the prominent X-zone in females serving as an additional signaling compartment, contributing to the overall higher communication complexity. The X/JMZ cluster, which is substantially larger in females, likely contributes disproportionately to the female communication network, particularly through predicted SPP1-integrin signaling. In males, the absence of a prominent X-zone may result in a less complex but more evenly distributed communication network across cortical zones. The subcapsular zona glomerulosa emerged as the dominant signaling hub in both sexes, but with markedly different activity levels and pathway engagement, likely reflecting its dual role as both a steroidogenic zone and a progenitor niche.

A central finding of this study is that sexual dimorphism in the adrenal cortex is not uniform but predominantly concentrated in inner cortical layers. The outer cortex, including the capsule, subcapsular zona glomerulosa, and zona glomerulosa proper, showed minimal sex differences in gene expression, while the inner cortex (outer ZF, middle ZF, inner ZF and X/JMZ) exhibited the most pronounced sexual dimorphism. It should be noted, however, that this spatial gradient is one of emphasis rather than absolute restriction: genes such as *Hmox1* display diffuse male-biased expression extending across multiple cortical zones, indicating that the outer cortex is not entirely exempt from sex-biased transcriptional regulation. This spatial gradient likely reflects differential hormonal regulation of inner versus outer cortical zones. The outer cortex, responsible for mineralocorticoid (aldosterone) production, is primarily regulated by the renin-angiotensin-aldosterone system (RAAS) and serum potassium, neither of which are sexually dimorphic under basal conditions^68,69^. In contrast, the zona fasciculata, which produces glucocorticoids (corticosterone in rodents, cortisol in humans), is regulated by the hypothalamic-pituitary-adrenal (HPA) axis, which exhibits well-documented sex differences in basal activity and stress responsiveness^17,33^. Female rodents generally display higher basal corticosterone levels, greater HPA axis reactivity to stress, and altered glucocorticoid receptor signaling compared to males, differences partially mediated by organizational and activation effects of gonadal steroids^30^. It is important to acknowledge, however, that designating the inner cortex as the primary locus of sexual dimorphism reflects the snapshot captured at a single developmental timepoint, 7-week-old sexually mature females (virgins). A recent bulk RNA-seq study^29^ profiling the mouse adrenal gland from postnatal day 0 through day 49 demonstrated that sex-dependent transcriptional differences emerge progressively throughout postnatal development, with divergence between male and female trajectories becoming pronounced from postnatal day 28 onwards. Genes associated with inner cortical identity (*Akr1c18*, *Pik3c2g*, *Spp1*) co-segregate in age- and sex-dependent co-expression modules, yet WNT pathway components and collagen-related genes exhibit age-dependent sex differences extending beyond the inner cortex. Together, these findings suggest that the inner cortex is a major, but not exclusive, contributor to adrenal sexual dimorphism, and that the full spatial extent of sex-biased gene expression is shaped dynamically by age and developmental stage. Our spatial atlas, by providing zone-resolved transcriptional data at a defined adult timepoint, offers a complementary and spatially explicit reference against which age-dependent transcriptomic trajectories can be anchored in future studies.

Our finding that steroid-metabolizing enzymes (*Akr1d1*, *Srd5a2*, *Cyp2f2*) are differentially expressed between sexes in the inner cortex suggests sex-specific post-synthetic modification of glucocorticoids^70^. 5α- and 5β-reductases convert active glucocorticoids to inactive metabolites (e.g., 5α-tetrahydrocorticosterone, 5β-tetrahydrocorticosterone), effectively terminating glucocorticoid signaling^71^. The distinct expression patterns of these enzymes between sexes may represent compensatory mechanisms to buffer sex-specific glucocorticoid production rates or receptor sensitivity.

Particularly intriguing is the X-zone-specific expression of *Srd5a2* in females, which contrasts with broad cortical expression in males. While 5α-reductase is best known for converting testosterone to the more potent androgen dihydrotestosterone (DHT) in peripheral tissues^72^, its role in the female adrenal X-zone-where androgen production is minimal-remains unclear. Possible functions include (i) local metabolism of adrenal-derived dehydroepiandrosterone (DHEA) or androstenedione, (ii) conversion of glucocorticoids or progesterone to neuroactive steroid metabolites, or (iii) regulation of steroid availability for export to peripheral tissues. Steroid metabolomics of X-zone tissue combined with functional studies using *Srd5a2* conditional knockout models will be necessary to resolve this question.

To ensure that sex-biased gene expression patterns identified by spatial transcriptomics reflect genuine biological differences, we performed immunohistochemical validation of selected markers (Fig. 7). The analysis confirmed coordinate regulation at both mRNA and protein levels, with striking validation of X-zone-specific patterns. The near-complete absence of AEBP1 and HMOX1 protein in the female X-zone, despite robust expression in the male corticomedullary region, confirms that transcriptional sex differences translate to functional protein-level divergence. HMOX1, an inducible stress-response enzyme that catalyzes heme degradation and generates cytoprotective carbon monoxide^73^, was one of the most male-biased genes in the X/JMZ cluster and showed concordant protein expression. However, it should be noted that both HMOX1 and AEBP1 immunostaining produced detectable signal in the medulla of both sexes, a pattern not reflected in the spatial transcriptomic data for either marker. Several non-mutually exclusive explanations may account for this discrepancy. First, antibody cross-reactivity cannot be excluded: both antibodies (anti-HO-1/HMOX1, 10701-1-AP; anti-AEBP1, HPA063595-25) are polyclonal reagents that may recognize epitopes shared with structurally related proteins expressed in chromaffin cells. Second, post-translational regulation, including protein stabilization, altered degradation rates, or stress-induced induction of HMOX1 in chromaffin cells, could result in protein accumulation disproportionate to steady-state mRNA levels. Third, HMOX1 is a well-characterized stress-inducible enzyme; its detection in medullary chromaffin cells may reflect constitutive low-level haem turnover activity not captured at the transcriptomic threshold applied in our analysis. Functional validation using independent antibodies, siRNA-mediated knockdown controls, or *Hmox1/Aebp1* knockout adrenal tissue will be necessary to resolve this issue definitively. Most strikingly, AKR1D1 immunohistochemistry revealed intense, sharply demarcated staining forming a distinct band in the female X-zone, with minimal expression in the male juxtamedullary zone, perfectly mirroring the transcriptomic data. The localization of AKR1D1 in the female X-zone suggests coordinated progesterone catabolism, resulting in complete inactivation of progesterone in virgin females^74^. It should be noted that the spatial extent of AKR1D1 protein expression was broader than the *Akr1c18* mRNA domain, suggesting incomplete overlap between mRNA and protein localization for this marker, an observation consistent with post-translational regulatory mechanisms or differential protein stability within the inner cortical compartment.The concordance between transcriptomic and immunohistochemical data validates the computational cell segmentation and cluster assignment approach employed in this study. The dramatic spatial segregation visible at the protein level, complete exclusion of male-biased proteins from the female X-zone, and precise restriction of female-biased steroidogenic enzymes to this region, are consistent with mechanisms maintaining distinct transcriptional programs through cell-autonomous factors or paracrine signals.

Several limitations should be acknowledged. First, our analysis captured a single timepoint (7-week-old sexually mature virgins under basal conditions), missing dynamic changes during estrous cycling, pregnancy, stress responses, and aging. The age of 7 weeks should be considered adolescent rather than fully adult, and sex-specific transcriptional patterns may differ in older animals. Future studies should include multiple age groups, spanning adolescence, adulthood, and senescence, to capture the full developmental trajectory of adrenal sexual dimorphism. Importantly, high-resolution spatial approaches will be indispensable for such analyses: whereas bulk RNA-seq or microarray methods applied to age- or sex-stratified cohorts can identify candidate zone-enriched genes, only spatial transcriptomics can reveal whether age-dependent transcriptional changes respect zonal boundaries, create novel subzonal gradients, or alter intercellular communication networks within specific cortical compartments. Second, transcriptomic predictions of steroid enzyme activities and cell-cell communication require validation through enzyme assays, steroid metabolomics,, functional receptor blockade, and genetic loss-of-function models. In particular, the predicted SPP1-integrin signaling axis in females should be functionally tested using *Spp1* knockout mice, which are viable and fertile, to determine whether SPP1 is required for X-zone maintenance. Third, mechanisms triggering X-zone involution during pregnancy remain unknown; time-resolved spatial transcriptomics across pregnancy stages combined with receptor knockouts will be necessary to identify the relevant signals. Fourth, while mice possess a prominent X-zone absent in rats and most species, and human adults lack an obvious X-zone equivalent despite a fetal/transitional zone that involutes postnatally. Application of Visium HD to human adrenal samples across sex, age, reproductive states, and disease conditions is essential for clinical translation. Fifth, the spatial trajectory analysis performed using SpaTrack should be interpreted with caution, as inferred trajectories reflect transcriptional similarity and spatial proximity rather than confirmed lineage relationships; true lineage tracing experiments will be required to determine whether the centrifugal transcriptional gradient observed along the JMZ/X-to-inner ZF axis reflects physical cell migration or zone-specific transcriptional states maintained by paracrine signaling. Additionally, pseudobulk differential expression and cell-cell communication analyses were performed on an unequal number of biological replicates per sex (*n* = 4 males, *n* = 2 females with complete adrenal cross-sections), as peripheral female sections lacking medullary and X-zone representation were excluded from these analyses. While the extreme magnitude of sex-biased gene expression in the X/JMZ cluster (log₂FC up to -16.28) and the consistency of spatial patterns across all sections support the robustness of our findings, the reduced female replicate number is a limitation that future studies should address by sectioning each adrenal gland individually. A further limitation inherent to spatial transcriptomic approaches using Cellpose-based cell segmentation is the potential for mis-clustering of small pockets of cells belonging to a lineage different from the surrounding tissue. In the adrenal gland, cortical steroidogenic cells are known to interdigitate within the medullary chromaffin cell mass; such cells may be assigned to the medulla cluster based on spatial proximity rather than true lineage identity. The scattered *Akr1c18*-positive signal observed within the medullary compartment in female spatial maps most likely reflects this phenomenon. Incorporation of lineage-specific markers such as *Nr5a1* (cortical steroidogenic cells) and *Th* or *Pnmt* (chromaffin cells) into future spatial panels would enable more precise delineation of cell identity at the corticomedullary boundary. Finally, gonadectomy studies with hormone replacement, combined with spatial transcriptomics, would help disentangle chromosomal versus hormonal contributions to sexual dimorphism. Despite these limitations, this spatial transcriptomic atlas provides unprecedented molecular resolution and establishes a foundation for future mechanistic and translational investigations.

## Conclusions

This study provides the first comprehensive, high-resolution spatial transcriptomic atlas of the sexually mature mouse adrenal gland, revealing pronounced sexual dimorphism in cellular organization, gene expression, and intercellular communication networks. The X-zone emerges as a molecularly distinct, female-enriched structure with unique steroidogenic capacity, including 20α-hydroxysteroid dehydrogenase-mediated progesterone catabolism and predicted paracrine signaling functions notably osteopontin/SPP1-mediated communication. Sexual dimorphism is most pronounced in the inner cortex, whereas the outer aldosterone-producing zones exhibit relatively limited transcriptional differences between sexes. However, selected transcripts such as *Hmox1* exhibit male-biased expression across broader cortical regions, indicating that sexual dimorphism spatially graded rather than strictly compartmentalized. Cell–cell communication analysis revealed sex-specific network organization, with females exhibiting greater overall signaling complexity, while also engaging distinct pathway-specific mechanisms. In particular, SPP1–integrin signaling is predicted to be enriched in the female X-zone microenvironment and represents a candidate paracrine pathway warranting future functional investigation. Spatial trajectory analysis reveals a dominant centripetal transcriptional gradient consistent with capsule-to-cortex differentiation, and a secondary centrifugal transcriptional axis emanating from the JMZ/X. This bidirectional organization, with convergence of capsule-derived and X-zone–derived transcriptional programs at the inner cortical boundary, is especially prominent in females and likely reflects the transcriptional activity of the X-zone. Together, these findings provide a molecular framework for understanding sex-specific adrenal physiology and pathophysiology, with potential implications for precision medicine approaches to adrenal disorders. The spatial transcriptomic resource generated in this study (accessible at https://adrenal-spatall-transcriptomic.shinyapps.io/adrenalsex/) offers a foundation for future investigations into adrenal development, stress responses, pregnancy-associated adaptations, disease mechanisms, and therapeutic targets in a sex-aware contex. As an essential reference for the adult adrenal gland, this atlas highlights the need for integrating spatially resolved transcriptomic datasets across developmental stages and sexes. Such efforts will be critical for understanding how age-dependent remodeling of zone-specific gene programs manifests at the level of individual cells and their microenvironmental niches.

Collectively, these insights underscore the importance of integrating spatial context, sex as a biological variable, and high-resolution transcriptomic profiling to advance both basic adrenal biology and the translation of discoveries into clinically actionable strategies.

## Materials and methods

### Ethical statement

All experimental procedures involving laboratory mice (*Mus musculus*) were conducted in compliance with the European Directive 2010/63/EU and the Polish Act on the Protection of Animals Used for Scientific or Educational Purposes. The experimental design, animal housing, and handling procedures were reviewed and approved by the Local Ethics Committee for Animal Studies in Poznan, Poland (approval no. 46/2025, issued on September 19, 2025). Animal health and welfare were monitored throughout the study by trained personnel. Daily assessments included observation of general activity, behavior, and grooming, as well as regular monitoring of food and water intake and stool consistency. All efforts were made to minimize animal distress and discomfort during the experimental procedures.

### Animals

Sexually mature BALB/c mice (*n* = 4 per sex), aged 7 weeks and weighing approximately 24 ± 2 g, were used in this study. Animals were bred and maintained at the Centre for Advanced Technology, Adam Mickiewicz University, Poznan, Poland. Mice were housed in colony cages under specific pathogen-free (SPF) conditions, with controlled temperature (21–23 °C), relative humidity (55–60%), and a 12-h light/dark cycle. Standard rodent chow and water were provided *ad libitum*. Animals were euthanized by decapitation between 9:00 and 10:00 am to minimize circadian variation in gene expression, and adrenal glands were immediately excised and fixed in 10% neutral buffered formalin for subsequent analyses.

### Tissue processing and Visium HD library preparation

Tissue specimens were formalin-fixed and paraffin-embedded (FFPE) following standard histological procedures. Tissue blocks were sectioned at 5 µm thickness using a Leica RM2255 microtome (Leica Biosystems, Wetzlar, Germany). FFPE sections were mounted onto SuperFrost™ Plus adhesion microscope slides (Fisher Scientific, Pittsburgh, PA, USA; catalogue no. 22-037-246) and air-dried at 42 °C, next slides were stored at 4 °C for further analysis. Adjacent serial sections were processed for RNA isolation using the RNeasy FFPE Kit (Qiagen, Hilden, Germany; catalogue no. 73504) to verify RNA yield and integrity. RNA quality was assessed using an Agilent 2100 Bioanalyzer (Agilent Technologies, Santa Clara, CA, USA), and only samples with DV200 > 50% were retained for downstream spatial transcriptomics analyses. Prior to spatial transcriptomics processing, slides were incubated at 60 °C for 2 h, followed by standard deparaffinization in xylene and rehydration through a graded ethanol series. Sections were subsequently stained with hematoxylin and eosin (H&E) using standard protocols and digitally scanned at 20× magnification with a Grundium Ocus 40 digital slide scanner (Grundium, Tampere, Finland). High-resolution brightfield images (~300 DPI) were acquired to ensure accurate alignment during Visium HD image registration and cell segmentation. Spatial transcriptomic profiling was performed using the 10x Genomics Visium HD Spatial Gene Expression workflow for FFPE samples with the CytAssist instrument, following the manufacturer’s instructions (10x Genomics, Pleasanton, CA, USA; User Guide CG000684, Rev B). The protocol included enzymatic permeabilization, RNase H-mediated probe cleavage, reverse transcription, and second-strand synthesis with spatially barcoded primers, followed by cDNA amplification. Spatially barcoded cDNA libraries were prepared according to the Visium HD Spatial Gene Expression Library Preparation protocol. Libraries from eight samples (four male and four female) were individually quantified using a Qubit fluorometer (Thermo Fisher Scientific, Waltham, MA, USA), normalized, and pooled in equimolar ratios. The pooled library was diluted to a final concentration of 400 pM in a total volume of 20 µL. Paired-end sequencing (2 × 150 bp) was performed on an Illumina Libraries were sequenced on an Illumina NovaSeq X Plus system Plus (Illumina, San Diego, CA, USA) using a 1.5B flow cell, with a 43–10–10–50 nucleotide configuration (Read 1 – i7 – i5 – Read 2) and a 1% PhiX spike-in, with 550 mln reads per capture area.

### Data processing and quality control

FASTQ files were inspected for sequencing depth parity between sexes. Initial quality assessment revealed a substantial imbalance in total sequencing output between male and female libraries (25.0 GB vs. 6.85 GB, ~3.65-fold difference). To eliminate this technical bias from downstream sex-comparative analyses, male FASTQ files were downsampled to match female sequencing depth using seqtk (version 1.3, downsample fraction = 0.274). Downsampling was applied consistently to all read types (R1, R2, I1, I2) across all sequencing lanes, with a fixed random seed (42) to ensure reproducibility. The downsampling script is publicly available at GitHub repository.

Individual cell boundaries were delineated from H&E-stained tissue images using Cellpose 3^41^, a deep learning-based segmentation algorithm. Prior to segmentation, high-resolution brightfield images were preprocessed with contrast-limited adaptive histogram equalization (CLAHE) applied to the L-channel in LAB color space to enhance cellular visibility in histological sections. Segmentation was performed using the generalist cytoplasm model (‘cyto3’) with GPU acceleration on an NVIDIA DGX Spark workstation. Due to the large size of whole-slide images, a tiled processing strategy was employed with 50-pixel overlap between adjacent tiles to ensure seamless cell detection across boundaries. Segmentation parameters were optimized for adrenal tissue morphology: estimated cell diameter of 40 pixels, flow threshold of 1.6, cell probability threshold of −4, and minimum cell size of 20 pixels. Segmentation performance was visually validated across representative tissue regions to ensure accurate cell boundary detection in morphologically distinct adrenal compartments. Segmentation masks were exported as NumPy arrays (.npy) with 32-bit unsigned integer encoding to accommodate the large number of detected cells per section.

Raw sequencing data (FASTQ files) were processed using Space Ranger version 4.0.1 (10x Genomics) installed on a local workstation. Read alignment, gene quantification, and spatial barcode assignment were performed against the *Mus musculus* reference genome (Genome Reference Consortium Mouse Build 38, GRCm38/mm10) with Ensembl gene annotations (release 2024-A). The Space Ranger pipeline encompassed adapter trimming, read alignment using STAR^75^, probe-target mapping for the Visium HD probe set, and unique molecular identifier (UMI) counting. Custom cell segmentation masks generated by Cellpose 3 were supplied to Space Ranger via the *--custom-segmentation* parameter, enabling transcript assignment at single-cell resolution based on morphologically defined cell boundaries. This approach allowed precise attribution of spatially barcoded transcripts to individual cells identified through deep learning-based image analysis. Final outputs included high-resolution tissue-aligned images with fiducial registration, cell boundary annotations, filtered feature-barcode matrices in HDF5 format (filtered_feature_bc_matrix.h5), spatial coordinate metadata (tissue_positions_list.parquet), and per-cell quality metrics. These outputs served as input for downstream bioinformatic analyses.

Processed data were imported into R (version 4.5.1)^76^ using the Seurat package (version 5.0.0)^77,78^. For each sample, count matrices were loaded with *Read10X_h5*, and Seurat objects were created using the “Spatial” assay. Spatial image data were imported using *Read10X_Image*, and high-resolution tissue images (*tissue_hires_image.png*) were integrated with appropriate scale factor adjustments to ensure accurate mapping of spatial coordinates. Quality control (QC) metrics included the number of UMIs per cell (*nCount_Spatial*), the number of detected genes per cell (*nFeature_Spatial*), and the percentage of mitochondrial gene counts (*percent.mt*). Initial QC and visual assessment were performed using violin plots (*VlnPlot*) and spatial feature plots (*SpatialFeaturePlot*) to identify potential technical artifacts, damaged tissue regions, or cells exhibiting abnormal transcriptional profiles.

### Data integration and normalization

Samples from male (*n* = 4) and female (*n* = 4) mice were merged into a single integrated Seurat object using the *merge* function. Following merge, data from multiple samples were stored in separate layers within the Seurat v5 object structure. These layers were consolidated using J*oinLayers* to create unified counts and data matrices across all samples. Data normalization was performed using the LogNormalize method with a scale factor of 10,000, which normalizes gene expression counts by total counts per cell. Highly variable genes (HVGs) were identified using the variance-stabilizing transformation (VST) method implemented in *FindVariableFeatures*, selecting the top 3000 features based on standardized variance. Data scaling was performed on HVGs using *ScaleData* to center expression values (mean = 0) and scale to unit variance (SD = 1), removing technical variation while preserving biological signal.

### Dimensionality reduction and batch correction

Principal component analysis (PCA) was performed on the scaled expression matrix of highly variable genes using *RunPCA*. The optimal number of principal components (PCs) retained for downstream analyses was determined using the elbow method by identifying the inflection (“knee”) point in the scree plot of standard deviations. PCs showing strong correlations with quality control metrics were excluded to minimize the influence of technical confounders. Batch correction and sample integration were carried out using Harmony (version 1.0)^79^ to align data across biological replicates while preserving genuine biological variation. Harmony was executed in the filtered PC space using *RunHarmony*, with grouping by sample identifier. The algorithm iteratively clusters cells and adjusts their embeddings to remove batch-specific effects while maintaining biologically meaningful structure.

### Clustering and cell type annotation

Unsupervised clustering was performed using the Louvain algorithm^80^ implemented in the *FindNeighbors* and *FindClusters* functions of the Seurat package. A shared nearest neighbor (SNN) graph was constructed in Harmony-integrated space using *FindNeighbors* with the final set of filtered dimensions and *k* = 20 nearest neighbors. Graph-based clustering was then conducted with *FindClusters* at multiple resolution parameters (1.0, 0.7, 0.5, 0.45) to evaluate cluster stability and determine the optimal level of granularity. Uniform Manifold Approximation and Projection (UMAP)^81^ was computed on the Harmony-corrected embeddings using *RunUMAP* to visualize cellular relationships in two dimensions. The clustering result obtained at a resolution of 0.45 was selected for downstream analyses. Cell type annotation was performed by assigning biological identities to clusters based on (i) the expression of established marker genes characteristic of adrenal cell populations, (ii) spatial localization patterns consistent with known adrenal gland architecture, and (iii) correlations with previously published single-cell and spatial transcriptomic datasets.

### Identification of cluster-specific marker genes

Cluster-specific marker genes were identified separately for male and female samples. To improve computational efficiency and reduce memory load, clusters were downsampled to a maximum of 4000 cells per cluster using random sampling. Differential expression analysis was performed using the Wilcoxon rank-sum test implemented in *FindAllMarkers*, with the following parameters: *only.pos* = TRUE (identifying only upregulated markers), *min.pct* = 0.20 (genes expressed in ≥20% of cells within a cluster), and *logfc.threshold* = 0.20 (minimum log₂ fold-change of 0.20). Statistical significance was determined using the Benjamini-Hochberg correction for multiple testing, and genes with an adjusted *p* < 0.05 were retained as significant cluster markers. For each cluster, the top five marker genes were selected based on average log₂ fold-change and visualized using heatmaps generated with *DoHeatmap*. Expression values were z-score normalized across genes, and hierarchical clustering was applied using Ward’s linkage method^82^.

### Sex-specific differential expression - analysis of the entire adrenal gland

To identify sex-biased gene expression across the entire adrenal gland, pseudobulk differential expression analysis was performed by aggregating single-cell UMI counts at the biological replicate level^83^. To ensure anatomically complete and balanced representation, only sections capturing the full adrenal cross-section including the medullary compartment and X-zone were included in pseudobulk analyses: four male sections (*n* = 4 biological replicates) and two female sections (*n* = 2 biological replicates). Peripheral sections lacking medullary representation were retained in the integrated atlas for cell type characterization and spatial visualization but excluded from sex-comparative differential expression and cell-cell communication analyses. For each replicate, UMI counts were summed across all spatial count layers to generate gene-by-replicate count matrices, where rows corresponded to genes and columns to biological replicates.

Genes were pre-filtered based on detection frequency: a gene was retained if expressed (UMI > 0) in ≥0.1% of cells in either males or females, thereby excluding lowly expressed genes unlikely to exhibit biologically meaningful differences. Differential expression testing was conducted using the edgeR package (version 3.40.0)^84,85^ within a quasi-likelihood F-test (QLF) framework. A design matrix was constructed with sex as a factor (female as the reference level), and the contrast tested was *Male - Female*. The analysis involved the following steps: (i) creation of a *DGEList* object, (ii) filtering of lowly expressed genes using *filterByExpr*, (iii) computation of normalization factors using the trimmed mean of M-values (TMM) method^86^, (iv) estimation of dispersion parameters with *estimateDisp*, (v) fitting of quasi-likelihood generalized linear models using *glmQLFit*, and (vi) statistical testing with *glmQLFTest*.

### Sex-specific differential expression analysis - Cluster-specific analysis

Sex-biased expression was further assessed within individual cell clusters using the same pseudobulk framework described above. For each cluster, cells were subset according to cluster identity, and UMI counts were aggregated per biological replicate within that cluster, generating cluster-specific gene-by-replicate count matrices.

Genes were pre-filtered based on cluster-specific detection: a gene was retained if expressed (UMI > 0) in ≥30% of cells in either males or females within the cluster. To further refine the analysis and focus on biologically relevant differences, testing was restricted to genes previously identified as cluster markers (from *FindAllMarkers*) in either sex. Differential expression analysis was then performed using the edgeR QLF framework as described for the whole-adrenal analysis.

### Cell-cell communication analysis with CellChat

Cell-cell communication networks were inferred separately for male and female adrenal samples (*n* = 4 male and *n* = 2 female biological replicates with complete adrenal cross-sections) using CellChat (version 2.2.0)^46^. Prior to analysis, peripheral adipose tissue clusters (WAT and BAT) were excluded to focus inference on adrenal-intrinsic signaling. To ensure fair statistical comparison between sexes and account for unequal cell numbers per cluster, balanced downsampling was performed prior to CellChat analysis. Individual CellChat objects were created from the downsampled Seurat objects containing normalized expression data (log-normalized counts, scale factor = 10,000) and cell type annotations. Spatial coordinates were extracted from Seurat metadata (centroid coordinates for segmented cells) and provided to CellChat to enable distance-based communication modeling. The mouse CellChat ligand-receptor interaction database (CellChatDB.mouse) was used, restricted to secreted signaling interactions (annotation = “Secreted Signaling”) to focus on paracrine and autocrine communication^46^.

### Spatial communication probability modeling

Communication probabilities were computed using a spatially constrained model that integrates both gene expression levels and physical distance between cells. Distance calibration was performed using k-nearest neighbor analysis (*k* = 5): the median nearest-neighbor distance (base_nn) was calculated, and an estimated cell diameter was defined as 1.5 × base_nn. Two spatial parameters were derived from this estimate: contact range (1.2 × estimated cell diameter), defining the threshold for direct cell-cell interactions; and interaction range (8.0 × estimated cell diameter), defining the maximum distance for biologically plausible paracrine signaling. The distance decay factor was set to 0.35. Communication probabilities were computed using the truncatedMean method (trim = 0.05) with full spatial constraints (distance.use = TRUE, contact.dependent = TRUE). To ensure statistical reliability, communications supported by fewer than 5 cells in either the sender or receiver population were filtered out (filterCommunication, min.cells = 5). Pathway-level communication probabilities were computed by aggregating all ligand-receptor pairs belonging to each signaling pathway (computeCommunProbPathway). The resulting interaction networks were aggregated across all pathways to generate summary statistics, including the total number of significant interactions (count matrix) and cumulative interaction strength (sum of communication probabilities, weight matrix)^87^.

### Comparative analysis between sexes

The two sex-specific CellChat objects were analyzed separately to preserve sex-specific network architectures. The two objects were subsequently merged using mergeCellChat for comparative visualization. Global communication metrics (total interaction count and aggregate strength) were compared between sexes using bar plots. Cell-to-cell communication networks were visualized using circle plots generated by netVisual_circle, where nodes represent cell populations and edges represent interactions. Line thickness represents the number of interactions between cell type pairs, and node size is proportional to the summed interaction strength for each population.

### Signaling pathway analysis

Differential signaling pathway activity between sexes was quantified by computing the log₂(Female/Male) ratio of total information flow (sum of communication probabilities across all sender-receiver pairs) for each pathway. Pathways were classified as female-enriched (log₂FC > 1), male-enriched (log₂FC < −1), or similar based on this ratio, and visualized as a ranked horizontal bar plot. Only pathways detected in at least one sex were included in visualizations.

### Spatial validation of predicted interactions

To validate computationally inferred signaling interactions, spatial gene expression patterns for key ligands and receptors were visualized using the original Visium HD spatial transcriptomics data. Expression levels for ligands and their cognate receptors were plotted on tissue spatial coordinates using the SpatialFeaturePlot function in Seurat. Co-localization of ligand and receptor expression was assessed by overlaying expression patterns in merged composite images to confirm spatial compatibility of predicted interactions. Spatial transcriptomic data based on bin-level centroid coordinates are publicly accessible via the interactive web atlas at https://adrenal-spatall-transcriptomic.shinyapps.io/adrenalsex/.

### Spatial trajectory analysis - data conversion and format preparation

For trajectory inference analysis and development of the interactive web application, Seurat objects^77^ were converted to AnnData format^88^ to ensure compatibility with Python-based spatial analysis tools. Conversion was performed in R, using the *SingleCellExperiment*^88^ and *zellkonverter*^89^ packages. Scaled expression data were extracted from the “Spatial” assay, and a *SingleCellExperiment* object was generated with cell metadata matched to barcode identifiers. Cluster identities were added to the *colData* slot, while dimensionality reduction embeddings (UMAP, PCA) and spatial coordinates were stored in *reducedDims*. The resulting *SingleCellExperiment* object was then exported to H5AD format for downstream analyses.

### Spatial trajectory inference with SpaTrack

Spatial trajectory analysis was performed using *SpaTrack*^90^ to reconstruct spatial developmental trajectories within the adrenal cortex. Analyses were conducted separately for male and female samples. Only clusters corresponding to the adrenal cortex and connective tissue capsule were included in the analysis.

### Data preprocessing

*AnnData* objects were loaded using scanpy (version 1.11.5)^91^. Spatial coordinates were verified and reformatted for compatibility with SpaTrack functions. Genes expressed in fewer than 10 cells were excluded prior to trajectory analysis. Expression values were normalized to 10,000 total counts per cell^92^. Quality control metrics, including the number of detected genes per spot and total counts per spot, were computed. The optimal transport (OT) matrix describing cell-to-cell transition probabilities was computed using *spt.get_ot_matrix* with the spatial data type^93,94^. This approach integrates transcriptomic similarity and spatial proximity through weighted parameters α₁ = 0.587 and α₂ = 0.413, empirically optimized for high-resolution Visium HD data. Principal component analysis was automatically performed when required, and the resulting transport matrix was stored in *adata.obsp[‘trans’]*.

### Pseudotime calculation and velocity estimation

Trajectory endpoint coordinates (START and END) were defined objectively for each sample as the spatial coordinates of the cell within the respective cluster nearest to the cluster centroid, ensuring reproducibility across samples. Pseudotime values were computed using the optimal transport (OT) matrix to quantify the relative progression of cells along the inferred spatial trajectory. Spatial velocity vectors were subsequently estimated using local neighborhoods of 50 cells to determine both the direction and magnitude of pseudotime progression across the tissue^95,96^. Two trajectories were analysed per sample: a centripetal trajectory from CT capsule to middle ZF (all 8 samples: M1–M4, F1–F4), and a centrifugal trajectory from JMZ/X to inner ZF (M1–M4 and F1–F2, restricted to samples with complete corticomedullary tissue representation).

### Identification of trajectory-dependent genes

To identify genes dynamically regulated along spatial trajectories, expression data were normalized and log-transformed. The expression matrix was converted to dense format, and non-finite values were replaced with zeros. Extreme expression values were clipped to prevent numerical instability during model fitting, and genes with zero total counts were excluded from further analysis. Trajectory-associated genes were filtered based on a minimum expression threshold (detected in ≥10% of cells) and high variability (top 5000 highly variable genes)^97^. Generalized Additive Models (GAMs) were fitted to test for significant associations between gene expression levels and pseudotime^98,99^. Genes with a model fit (*R²*) > 0.15 and a false discovery rate (FDR) < 0.05 (Benjamini-Hochberg correction) were considered significantly associated with the trajectory. For cross-sex comparisons, genes consistently identified in ≥2 independent biological replicates of the same sex were included. Mean expression per pseudotime bin (50 bins) was calculated across replicates and min-max normalized per gene prior to visualization.

### Interactive web application for data exploration

To facilitate broad access to the spatial transcriptomic dataset and enable interactive exploration, a web-based visualization platform was developed and deployed. The application provides real-time, interactive visualization of gene expression patterns across spatial, UMAP, and statistical dimensions for both male and female mouse adrenal glands. The interactive atlas was built using Plotly Dash^100^ and containerized with Docker^101^ to ensure reproducible deployment. The platform consists of a single-page dashboard with multiple interactive tabs, each providing complementary perspectives on the spatial transcriptomic data. Data originate from preprocessed *H5AD* files containing normalized expression matrices, dimensionality reductions (UMAP, PCA), spatial coordinates, and cluster annotations for male and female samples separately. The Docker container was built using Python (version 3.10) and included the following dependencies: *scanpy*^91^, *anndata*^88^, *pandas*^102^, *numpy*^103^, *plotly*^100^, *dash*, *dash-bootstrap-components*, *scikit-learn*^104^, *scipy*^105^, and *Pillow*. The application was deployed behind a reverse proxy (Caddy) on a shared frontend network, enabling secure HTTPS access and seamless integration with existing infrastructure. The web application comprises five main interactive modules, accessible via navigation tabs: (1) Spatial visualization: Displays gene expression overlaid on H&E-stained tissue images for male and female adrenal glands side-by-side, using faceted scatter plots. Users can select any gene from a searchable dropdown list (19,070 genes available) and adjust visualization parameters including point size (1–50 pixels), opacity (0.1–1.0), and color scale (Viridis, Plasma, Cividis, Turbo, or transparent gradients). Expression levels are displayed as continuous color gradients with a shared colorbar. (2) UMAP projection: Projects gene expression onto a joint UMAP embedding computed from combined male and female datasets. Expression is visualized as a continuous color gradient (Viridis scale) overlaid on 2D UMAP coordinates. Cluster centroids are annotated with text labels. Point size is fixed at 3 pixels with 80% opacity. (3) Violin plots: Compares gene expression distributions across clusters, stratified by sex. Violin plots are generated using *plotly express*, with overlaid box plots and individual data points. Separate violins are displayed for male (blue) and female (red) samples within each cluster, allowing direct comparison of sex-specific expression patterns and variability. (4) Heatmap visualization: Displays hierarchical clustering of top marker genes across cell populations. Users can select the number of top marker genes per cluster (2–20, default = 5) using a slider. Markers are ranked by average log₂ fold-change, and the top *N* genes per cluster are extracted. Expression values are aggregated by computing the cluster-wise mean and subsequently z-score normalized across genes^104^. Hierarchical clustering (Ward’s method) is applied to both rows (clusters) and columns (genes) using *scipy*^105^. (5) Multiple gene overlay: Enables simultaneous visualization of up to four genes with distinct color channels (green, red, blue, yellow) overlaid on spatial coordinates. Users can select four genes from dropdown menus and adjust point size and opacity. Each gene’s expression is rendered as a semi-transparent gradient, allowing visualization of co-localization patterns through additive color mixing. Male and female tissues are displayed side-by-side on H&E backgrounds. This module uses *Scattergl* (WebGL-accelerated rendering) to ensure high-performance visualization of large datasets. All data are freely accessible through an interactive web application at https://adrenal-spatall-transcriptomic.shinyapps.io/adrenalsex/.

### Immunohistochemical staining

Tissue section staining of FFPE blocks was carried out following previously established protocols^106–108^. Briefly, after deparaffinization, tissue sections were rehydrated through a graded ethanol series of decreasing concentrations and rinsed in phosphate-buffered saline (PBS). Heat-induced epitope retrieval (HIER) was then applied to expose antigenic sites for DAB2 detection. Slides were immersed in Target Retrieval Solution, Citrate pH 6.1 (#H-3300-250, Vector Laboratories, Stuttgart, Germany) and heated in a microwave oven for 5 min, followed by cooling at room temperature for 20 min. Endogenous peroxidase activity was quenched, and nonspecific binding was blocked using 2.5% normal horse serum. The sections were then incubated overnight at 4 °C with the appropriate primary antibody, as detailed in Table 1. After washing, signal detection was performed using the ImmPRESS® HRP Universal (Horse Anti-Mouse/Rabbit IgG) PLUS Polymer Kit (#MP-7800, Vector Laboratories, Stuttgart, Germany) according to the manufacturer’s instructions. Slides were counterstained with Mayer’s hematoxylin (#S330930-2, Dako, Glostrup, Denmark), dehydrated, and mounted. Finally, digital slide images were captured using a Grundium Ocus® 40 microscope scanner (Grundium Ltd., Tampere, Finland).

**Table 1:** Primary antibodies and dilutions used for immunohistochemical analysis of adrenal tissue

Antibody name	Catalog number	Dilution	Company
Anti-HO-1/HMOX1	10701-1-AP	1:500	Proteintech, (Rosemont, IL, USA)
Anti-AEBP1	HPA063595-25	1:100	Atlas Antibodies AB (Bromma, Sweden)
Anti-NREP	HPA000545-25	1:100	
Anti-AKR1D1	HPA057002-100	1:50	

### Reporting summary

Further information on research design is available in the Nature Portfolio Reporting Summary linked to this article.

## Supplementary information

Transparent Peer Review file

Supplementary Information

Supplementary Data 1

Supplementary Data 2

Reporting Summary

## Data Availability

All datasets have been deposited in the Gene Expression Omnibus (GEO) database under accession number GSE312015^109^. https://adrenal-spatall-transcriptomic.shinyapps.io/adrenalsex/ All data supporting the findings of this study are available within the article, its Supplementary Information files, and Supplementary Data 1 and 2.

## Code availability

Code used for data analysis has been deposited at GitHub (https://github.com/MarcinRuc78/adrenal_male_female).
