# Supplementary material for: Single-cell spatial transcriptomics reveals sex-dependent gene expression and intercellular signalling in mouse adrenal cortex
Source: Commun Biol. 2026 Jul 14;9:1480. doi: 10.1038/s42003-026-10697-9 (PMC13740276; doi:10.1038/s42003-026-10697-9)
Supplement: Supplementary file 1 [file 42003_2026_10697_MOESM1_ESM.pdf]

nature portfolio

Peer Review File

Single-cell spatial transcriptomics reveals sex-dependent gene expression and intercellular signalling in mouse adrenal cortex

Corresponding Author: Dr Małgorzata Blatkiewicz

This file contains all reviewer reports in order by version, followed by all author rebuttals in order by version.

Version 0:

Reviewer comments:

Reviewer #1

(Remarks to the Author)
The reviewer considers this to be an important reference dataset for future adrenal gland research especially for studies using the mouse as an animal model.

The authors appropriately cite multiple related studies to support individual statements in the text, which helps strengthen the justification and acknowledge prior work. However, this citation practice is not applied consistently throughout the manuscript. Some earlier foundational studies should be included, rather than relying predominantly on more recent articles published in high-impact journals. For example, in the first two sentences of the third paragraph of the Introduction, the authors cite four and five references, respectively. In contrast, for several key findings in the field—such as early lineage-tracing studies identifying stem/progenitor markers—important original work is not cited. The authors should review the introduction and the discussion sections to ensure that seminal studies are appropriately acknowledged.

Results, Section 1: Although methodological details are provided in the Materials and Methods section, clarity would be improved by specifying the mouse strain and age in the first sentence of the Results section, rather than referring only to "sexually mature mice." Because sexual dimorphism is known to be age- and strain-dependent, the authors may also consider briefly addressing this concept in the Discussion.

Discussion: As discussed in the second paragraph of the first Discussion section, the concentric zone was discovered based on bulk RNA-seq and IHC. It would be helpful if the authors could emphasize that, prior to the development of spatial transcriptomics, bulk RNA-seq or microarray approaches (both bulk-level omics methods) already had the potential to identify novel zone-specific genes in the adrenal gland by comparing glands across different conditions, such as sex or age. The authors should also highlight the importance of spatial approaches for future studies of adrenal glands at different ages, and discuss how age- and sex-dependent spatial transcriptional data can serve as a valuable reference to shape the future of adrenal gland research.

In the last two sections of the Discussion, the authors highlight the important role of the inner cortex in sexual dimorphism. The reviewer agrees with this focus, as it reflects a long-standing understanding in the field. However, the editor notes that this spatial omics study further supports a recently published bulk RNA-seq study showing that sexual dimorphism is a dynamic, age-dependent process. Together, these findings suggest that the inner cortex, although potentially a major contributor, is not the sole region responsible for sexual dimorphism in the mouse adrenal gland. The reviewer suggests that the authors include this concept in the Discussion section.

Reviewer #2

(Remarks to the Author)
In this study, the authors performed spatial transcriptomic analyses using Visium HD technology to identify differentially expressed genes and their spatial distribution across adrenal cortical zones and between sexes. Their analyses identified 136 sexually dimorphic genes, with the majority being female-specific, largely attributable to the persistence of the X-zone in females. Moreover, bioinformatic analyses suggested enhanced Sonic hedgehog (Shh) signaling in males, whereas

increased SPP1–integrin signaling was predicted in females. Spatial trajectory analysis using SpaTrack was applied to infer differentiation pathways and potentially define new ones. Finally, expression analyses using five different antibodies were conducted in an attempt to validate findings derived from the bioinformatic analyses.

Understanding the basis of sexual dimorphism in non-reproductive organs could shed light on sex-specific physiology and disease susceptibility. As such, this manuscript addresses a relevant and timely topic. However, the study is largely descriptive and many of the conclusions are drawn from bioinformatic analyses without functional validation. Consequently, the manuscript can be considered a resource rather than a basic research paper. As the vast majority of the findings are predictions, the claims are overly strong and should be toned down. Furthermore, I believe that achieving the level of impact appropriate for a Communications Biology publication would require additional experimental evidence.

Specific points:
1. According to the Materials and Methods section, libraries were prepared from sections derived from four male and four female adrenals; however, apart from Fig. 1C/D, which show quite a bit of difference between sections (e.g. subcapsular populations), the authors present only merged datasets. It would be important to provide at least one supplementary figure showing U-Maps and cell distribution in individual samples to demonstrate consistency and reproducibility across biological replicates.
2. Figure 2: Srd5a2 shows sexually dimorphic expression, with expression throughout the cortex in males but restricted to the X-zone in females. I assume that the images in Figure 2B represent graphical outputs of the Visium HD data. Are these scaled independently for each sex? In other words, are expression levels in the female X-zone directly comparable to those observed in the male cortex? This point should be clarified perhaps by using violin plots to provide actual read data. Ideally, this observation should be confirmed using RNA-scope analysis.
3. The reported higher expression of Shh in males is unexpected in particular when considering the higher recruitment of Gli1 positive capsular cells in females. Can this be confirmed using other samples (qPCR/ RNA-Scope)? Also, the sexually dimorphic expression of Itgav should be experimentally validated given that good antibodies are available.
4. Spatial trajectory analysis: This type of analysis predicts ‘potential’ trajectories with the assumption that cells are differentiating. However, as the authors rightly point out in the discussion, transcriptomes may also change along signalling gradients without a true trajectory. As there are presently no true lineage tracing data for the X/JMZ zone the paragraph on bidirectional flow remains largely speculative and the title “Spatial trajectory inference reveals bidirectional cell flow’ needs to be changed.
5. The heatmaps of trajectory associated genes in Figure 7B appear to be different for male and female samples. This is surprising given that centripetal migration occurs in both sexes. Could the authors please show the heatmaps for male and female trajectory associated genes for both sexes? What does the ‘trajectory’ for the X/JMZ look like? Does it provide any meaningful clues regarding the transcriptional changes along the centrifugal axes? Are they convincing in all 4 female samples analysed?
6. Osteopontin as a mechanism to maintain the X-zone: Osteopontin expression in the female X-zone has been previously reported. While SpaTrack predicts interactions with integrins, the presented data do not demonstrate that X-zone maintenance is mediated through this pathway (as claimed in the discussion). Ideally, analysis of adrenal tissue from Spp1 knockout mice should be analyzed (these ko mice survive and are fertile) to provide functional data and to support this hypothesis. If such experiments are not feasible, the claim of osteopontin signaling as a mechanism of maintaining the X-Zone in females should be rewritten and shortened to reflect the speculative nature of the findings.
7. Figure 8: HMOX1 and AEBP1 immunostaining shows strong signals in the medulla, whereas these cells appear negative in the spatial transcriptomic analysis. This discrepancy raises concerns regarding antibody specificity, which should be addressed.
The Discussion section is rather long and contains several claims that lack sufficient experimental support:
8. The statement that “this study presents the first comprehensive high-resolution spatial transcriptomic … revealing profound sexual dimorphism that is spatially restricted to inner cortical zones” is very strong. Doesn't the authors’ own data suggest that Hmox1 is sexually dimorphic throughout the cortex?
9. The X-zone–specific expression of Akr1c18 (and its female specificity) was reported several decades ago. This section of the Discussion should therefore be substantially shortened.
10. Paragraph: “Sex differences in intercellular communication networks: complexity versus pathway specificity.” Bioinformatic predictions of pathway activity are inherently challenging and should generally be viewed as a basis for future experimentation. Gene expression alone does not necessarily imply pathway activation or biological relevance in this context. Statements such as “the X-zone serves as a centralized signalling hub (as evidenced by SPP1)” are therefore not sufficiently supported and should be revised.
Minor points
9. First paragraph: The statement “We obtained ~104,000 high-quality single-cell transcriptomes” is misleading. These data represent inferred single cells derived from spatial transcriptomics, which does not allow unambiguous single-cell resolution. This should be clarified.
10. Experimental setup: The authors used 7-week-old mice, which should be considered adolescent rather than fully adult. This limitation should be explicitly acknowledged.
11. Discussion, page 17: The sentence “we performed immunohistochemical validation of selected markers (Figure 9)” should refer to Figure 8, not Figure 9.

Reviewer #3

(Remarks to the Author)
The adrenal gland is recognized as one of the most sexually dimorphic organs. Understanding sex-dependent mechanisms

in adrenal gland function is critical, as many adrenal diseases occur more frequently in females. Despite differences in structure and steroid production between humans and rodents, they share many similarities, and transgenic mouse models have proven useful for deciphering molecular mechanisms in adrenal development and pathology.
In the present manuscript entitled ‘Single-cell spatial transcriptomics reveals sex-dependent gene expression and intercellular signaling in mouse adrenal cortex’, Blatkiewicz and colleagues employ Visium HD spatial transcriptomics to profile transcriptomes from 7-week-old male and female adrenals and explore sex-dependent mechanisms in the adrenal gland. The authors present a valuable accessible resource, which is appreciated. However, some aspects require clarification, some figures would benefit from reorganization, and some statements need to be softened. Nevertheless, once revised, this manuscript is expected to provide critical information for the scientific community and has the potential to be well cited. Below are our detailed comments.
Major comments:
1) Line 37: The claim that ‘females engage SPP1-integrin pathways for X-zone maintenance’ requires experimental validation. Such a conclusion should be rephrased (i.e., the data suggests...) unless functional experiments are provided (for example, Spp1 KO model).
2) Akr1c18 as a ‘definitive marker’ (Line 35) or ‘gold-standard’ (Line 498) is debatable. Indeed, mouse models such as Prkar1a KO and Hhex KO, with increased Akr1c18 expression in males despite the absence of X-zone and Pik3c2g expression, suggest that Akr1c18 can be expressed in cells of the definitive lineage. Perhaps the authors could soften the conclusion or focus on Pik3c2g, which seems to be a consistent X-zone marker across studies and mouse models.
3) Line 38, I would recommend choosing a different word than ‘establish’ since the X-zone capacity to metabolize progesterone has been demonstrated since 2007 at least (Hershkovitz et al., cited line 491).
4) Line 65, the statement that the ‘molecular basis (of adrenal sexual dimorphism) remains poorly understood’ is not accurate. There has been considerable characterization over the last decade, particularly implicating the androgen receptor (AR), its target gene Hhex, Wnt signaling, senescence, immune response etc…. Please revise to acknowledge and provide the reader with the most recent existing publications. Among few examples: Dolfi et al., 2022. Unravelling the sex-specific diversity and functions of adrenal gland macrophages. Wang et al., 2025. The Transcriptome Trajectory Reveals Sex- and Age-Dependent Changes in the Mouse Adrenal Gland, Takahashi et al., 2024, Development of sexual dimorphism of skeletal muscles through the adrenal cortex, caused by androgen-induced global gene suppression., Dumontet et al. 2026, The transcription factor HHEX maintains glucocorticoid levels and protects adrenals from androgen-induced lipid depletion. Grabek and al., 2019 demonstrated that females employ an additional stem and/or progenitor compartment. This information is lacking in the introduction, while it could perhaps support the analysis the authors performed in Figure 5D.
5) Perhaps the authors could clarify the knowledge gap they address in the present manuscript. Is it about the X-zone, the cell-cell communication, etc..? I recommend highlighting more the accompanying web app for data visualization, both in the abstract and the introduction.
6) Figure 5 and 6 could be organized together for better continuity, as well as 3 & 4. Figure 8 should accompany figure 4, the current organization is confusing.
7) Figure 3H and Figure 4. The increased expression of Akr1c18 in the medulla is likely due to steroidogenic cells localized among medulla cells. Perhaps the authors could add Sf1 to their panel to assess this. If accurate, could the authors comment on the spatial method and its limitations in regard to misidentifying/mis-clustering small pockets of cells belonging to a different lineage than the tissue in which they are found?
8) In the results section, it would be valuable to briefly introduce the functions of genes listed. For example, Susd3 and Ddx3y are not mentioned in the text, yet the authors show in Figure 4 that they are sexually dimorphic in the zG and outer zF. I suggest these two genes, Mt1 and mt-Nd4 be added to the panel of spatial transcriptomic maps as well. Aebp1 function is also not mentioned, while the authors put efforts into validating this gene by immunohistochemistry.
9) Line 408: The authors describe AKR1D1 expression domain as a ‘sharply demarcated band at the corticomedullary boundary in females corresponding precisely to the X-zone’, but the spatial maps presented in Figure 4 showed a clear wider expression pattern than Akr1c18. Are the authors suggesting that mRNA and protein expression are incompletely overlapping?
10) In regards of Per1 pattern in the trajectory analysis, were all the samples collected at the same timepoint? If yes, provide this information in the methods section and regard the strong circadian rhythmicity of Per1 expression.
11) CYP2F1 data are confusing. I suggest removing it or explaining the rationale for the analysis and the conclusion it supports in greater detail. Is this piece of data related to CYP2F2 expression in the inner zF by any means?
12) Line 417: ‘These findings confirm that sexual dimorphism in the mouse adrenal gland is spatially restricted to the corticomedullary boundary’. This statement contradicts Figure 4: Hhex, Acsbg1, Mgarp, and Jun are clearly sexually dimorphic and not restricted to the corticomedullary boundary. I suggest revising the conclusions or clarifying their meaning.
13) From lines 337 to 384: The spatial trajectory analysis is interesting, and the authors suggest bidirectional flow. However, the second trajectory originates from the X/JMX, but the GAM analysis seems to be missing for this trajectory.

Minor comments:
1) Line 35, statistical details in the abstract are unnecessary and could be restricted to the results and method sections.
2) Add the age in the results section and the legend of the figure (n=4 males, 4 females, 7 week-old).
3) Line 314: Figure 6A and 6B are flipped in the result section.
4) Scales are missing from all histology pictures.

Typos:
1) Line 100: in the introduction: ‘spatial restriction’
2) Line 102: Specialized in progesterone catabolism instead of specialized that progesterone catabolism

Reviewer #4

(Remarks to the Author)
I co-reviewed this manuscript with one of the reviewers who provided the listed reports. This is part of the Communications Biology initiative to facilitate training in peer review and to provide appropriate recognition for Early Career Researchers who co-review manuscripts.

Version 1:

Reviewer comments:

Reviewer #1

(Remarks to the Author)
This reviewer has no additional comments. This dataset will be a valuable resource for the adrenal research community.

Reviewer #2

(Remarks to the Author)
The authors have taken most of my comments into account and the more careful wording is now acceptable. I appreciate the new analysis using data that were adjusted for sequencing depth, and the use of the Cellpose 3 algorithm. Additional experiments to support their hypothesis on the osteopontin/SPP1 axis would have been nice, but perhaps this is beyond the scope of this paper.

Minor point:
Please double check the official nomenclature (e.g. I spotted a couple of places were knockouts were not labelled in italic):
Mouse protein: SHH (Capital, straight)
Mouse gene, RNA, and knockouts: Shh (Only first letter capital, gene name in italic)

Reviewer #3

(Remarks to the Author)
I read the answers provided by the authors to the reviewers comments and my major concerns are:

The functional validation requested by the reviewers have been declined by the authors, including validation of SPP1 pathway at the protein level which is now the main point of the current manuscript, after removal of Shh expression data. Overall, the current manuscript is an important technical innovation for the field, but the results presented are not novel or strongly supported by functional analysis/orthogonal approaches.

It appears that the dataset has been drastically reanalyzed. According to the authors, this was in part to correct a technical bias. As a result, conclusions of the manuscript have been altered, including the sexually dimorphic expression of Shh. According to the authors, this 'reflected a preliminary result that did not survive more rigorous analytical framework'. Another concern is the use of antibodies with cross-reactivity that might be 'insufficient for reliable detection under the conditions employed' according to the authors. These statements alone raise significant concern regarding the scientific rigor. The staining for CYP2F1 has no value in the current manuscript and do not replace staining for Cyp2f2, antibodies suitable for IHC are available and already been published (Lyu et al 2020, endocrinology)

Additional comments: potential panel duplication between figure 2 and figure 3 (abcb1b, akr1b7)

Reviewer #4

(Remarks to the Author)
I co-reviewed this manuscript with one of the reviewers who provided the listed reports. This is part of the Communications Biology initiative to facilitate training in peer review and to provide appropriate recognition for Early Career Researchers who co-review manuscripts.

Version 2:

Reviewer comments:

Reviewer #3

(Remarks to the Author)
I do not have additional comments. The manuscript has been significantly improved, and the data set will be an important resource for the scientific community.

Reviewer #4

(Remarks to the Author)

I co-reviewed this manuscript with one of the reviewers who provided the listed reports. This is part of the Communications Biology initiative to facilitate training in peer review and to provide appropriate recognition for Early Career Researchers who co-review manuscripts.

Open Access This Peer Review File is licensed under a Creative Commons Attribution 4.0 International License, which permits use, sharing, adaptation, distribution and reproduction in any medium or format, as long as you give appropriate credit to the original author(s) and the source, provide a link to the Creative Commons license, and indicate if changes were made.
In cases where reviewers are anonymous, credit should be given to 'Anonymous Referee' and the source.
The images or other third party material in this Peer Review File are included in the article's Creative Commons license, unless indicated otherwise in a credit line to the material. If material is not included in the article's Creative Commons license and your intended use is not permitted by statutory regulation or exceeds the permitted use, you will need to obtain permission directly from the copyright holder.
To view a copy of this license, visit https://creativecommons.org/licenses/by/4.0/

Reviewers' comments:

Reviewer #1 (Remarks to the Author):

The reviewer considers this to be an important reference dataset for future adrenal gland research especially for studies using the mouse as an animal model.

We thank the reviewer for this positive assessment. We would like to highlight two important methodological improvements that further strengthen the value of this dataset.

First, we have re-analyzed all samples using Cellpose 3 (cyto3 model) for cell segmentation, replacing the default Space Ranger segmentation approach. The default Space Ranger pipeline performs nuclear detection followed by cytoplasmic expansion, which results in exclusion of cells whose cross-sections do not contain a nucleus in the tissue section plane - a particularly significant limitation in adrenal tissue sections where many steroidogenic cells are large and the nuclear plane may not be captured. Cellpose 3 performs whole-cell boundary detection directly from H&E morphology, recovering a substantially larger number of cells and providing more accurate cell boundary delineation. The resulting segmentation masks (.npy format) were supplied to Space Ranger via the --custom-segmentation parameter, enabling transcript assignment at true cell resolution. This re-analysis increased the total number of recovered cells from the previously reported estimate to 203,069 cells across all eight samples (104,843 male, 98,226 female), substantially improving the depth and reliability of the atlas.

Second, while centroid-based coordinates were used for cell-cell communication analysis (CellChat) and are available through the interactive web atlas, full cell boundary segmentation masks are now utilized for spatial visualization within the Seurat framework (version 5.4.0), which natively supports cell boundary rendering. This allows visualization of gene expression at true single-cell resolution with accurate cell outlines rather than point-based representations, as implemented in the updated figures.

Additionally, to ensure unbiased sex-comparative analyses, we identified a substantial imbalance in sequencing depth between male and female libraries (25.0 GB vs. 6.85 GB total FASTQ output, ~3.65-fold difference). To correct for this technical bias, male FASTQ files were downsampled to match female sequencing depth prior to Space Ranger processing using seqtk (downsample fraction = 0.274, random seed = 42), and all analyses presented in this manuscript were performed on the depth-balanced dataset. We note that the original, undownsampled FASTQ files - including the higher-depth male libraries - have been deposited in GEO alongside the processed data, allowing future users to reprocess the data with alternative normalization strategies or to take advantage of the additional sequencing depth for male-specific analyses where sex comparison is not the primary objective.

The authors appropriately cite multiple related studies to support individual statements in the text, which helps strengthen the justification and acknowledge prior work. However, this citation practice is not applied consistently throughout the manuscript. Some earlier foundational studies should be included, rather than relying predominantly on more recent articles published in high-impact journals. For example, in the first two sentences of the third paragraph of the Introduction, the authors cite four and five references, respectively. In contrast, for several key findings in the field—such as early lineage-tracing studies identifying stem/progenitor markers—important original work is not cited. The authors should review the introduction and the discussion sections to ensure that seminal studies are appropriately acknowledged.

We thank the Reviewer for this important observation regarding citation consistency. We agree that the manuscript showed uneven citation practice, with some statements supported by multiple references while key foundational studies, particularly in the lineage tracing and stem/progenitor cell literature, were not individually attributed.

We have reviewed both the Introduction and Discussion sections with this concern in mind and introduced the following corrections.

Results, Section 1: Although methodological details are provided in the Materials and Methods section, clarity would be improved by specifying the mouse strain and age in the first sentence of the Results section, rather than referring only to “sexually mature mice.” Because sexual dimorphism is known to be age- and strain-dependent, the authors may also consider briefly addressing this concept in the Discussion.

We thank the reviewer for this suggestion. We have revised the first sentence of the Results section to explicitly state the mouse strain and age:
"To comprehensively characterize the cellular composition and spatial organization of the mouse adrenal gland, we performed high-resolution spatial transcriptomics on adrenal tissues from 7-week-old BALB/c mice (n = 4 males, 4 females)."
Regarding the age- and strain-dependence of sexual dimorphism, we have added the following statement to the Limitations section of the Discussion:
"The age of 7 weeks should be considered adolescent rather than fully adult, and sex-specific transcriptional patterns may differ in older animals; future studies should include multiple age groups to capture the full developmental trajectory of adrenal sexual dimorphism."

Discussion: As discussed in the second paragraph of the first Discussion section, the concentric zone was discovered based on bulk RNA-seq and IHC. It would be helpful if the authors could emphasize that, prior to the development of spatial transcriptomics, bulk RNA-seq or microarray approaches (both bulk-level omics methods) already had the potential to identify novel zone-specific genes in the adrenal gland by comparing glands across different conditions, such as sex or age. The authors should also highlight the importance of spatial approaches for future studies of adrenal glands at different ages, and discuss how age- and sex-dependent spatial transcriptional data can serve as a valuable reference to shape the future of adrenal gland research.

We thank the Reviewer for this insightful comment, which prompted us to more explicitly acknowledge the foundational contributions of bulk transcriptomic approaches and to better articulate the forward-looking value of spatial transcriptomics for age- and sex-stratified adrenal research.
We have introduced two targeted additions to the manuscript:
1. Second paragraph of the first Discussion section (following the sentence describing Lyu et al.'s identification of ZF sub-zones based on RNA-seq and IHC):
"This illustrates that bulk-level transcriptomic approaches, including RNA-seq and microarray analysis, already held the potential to reveal novel zone-specific molecular signatures in the adrenal gland when applied to comparisons across physiological conditions such as sex or age — yet without spatial resolution, the precise anatomical boundaries of these molecular domains could not be established."
This addition explicitly recognises that bulk-level omics methods were instrumental in identifying candidate zone-specific genes prior to the advent of spatial transcriptomics, while clarifying that spatial resolution was the critical missing dimension that prevented full characterisation of their anatomical context.
2. Conclusions section (inserted between the sentence describing the Shiny App resource and the closing synthesis sentence):
"This resource represents an essential baseline reference for the adult sexually mature adrenal gland; integrating spatially resolved transcriptomic atlases across multiple ages and sexes will be critical for dissecting how age-dependent remodelling of zone-specific gene programmes — already revealed by bulk RNA-seq and microarray studies comparing adrenal glands across sex or age — manifests at the level of individual cells and their microenvironmental niches."
This addition directly addresses the Reviewer's request to highlight both the importance of spatial approaches for future age-stratified studies and the value of age- and sex-dependent spatial transcriptional data as a reference framework for the field.

In the last two sections of the Discussion, the authors highlight the important role of the inner cortex in sexual dimorphism. The reviewer agrees with this focus, as it reflects a long-standing understanding in the field. However, the editor notes that this spatial omics study further supports a recently published bulk RNA-seq study showing that sexual dimorphism is a dynamic, age-dependent process. Together, these findings suggest that the inner cortex, although potentially a major contributor, is not the sole region responsible for sexual dimorphism in the mouse adrenal gland. The reviewer suggests that the authors include this concept in the Discussion section.

We thank the Reviewer for drawing our attention to this important conceptual point. We agree that framing sexual dimorphism exclusively in terms of inner cortical dominance risks oversimplification, and that the emerging picture, particularly in light of recent transcriptomic evidence, is one of a dynamic, spatially and temporally distributed phenomenon.

We note that the Reviewer likely refers to the recent study by Wang et al. (2025, *Aging Cell*, PMID: 40650660), which profiled the mouse adrenal gland transcriptome across postnatal days 0, 7, 15, 21, 28, 35, and 49 using bulk RNA-seq. That study demonstrated that sex-dependent differences in gene expression increase progressively over postnatal development, with male and female transcriptomic trajectories beginning to diverge markedly from postnatal day 28 onwards, coinciding with the pubertal androgen surge. Notably, Wang et al. identified zone-specific inner cortex markers (*Akr1c18*, *Pik3c2g*, *Cyp2f2*, *Spp1*, *Frzb*) as clustered within the same co-expression module, and further showed that WNT signalling components exhibit age-dependent sex differences across the whole gland, extending beyond the inner cortex per se.
In light of this, we have added the following passage to the section "Spatial restriction of sexual dimorphism to inner cortical zones", at the end of the second paragraph of that section (after the discussion of HPA axis sex differences). We believe this addition directly addresses the Reviewer's suggestion and appropriately contextualises our findings within the broader, dynamically evolving picture of adrenal sexual dimorphism.

Reviewer #2 (Remarks to the Author):

In this study, the authors performed spatial transcriptomic analyses using Visium HD technology to identify differentially expressed genes and their spatial distribution across adrenal cortical zones and between sexes. Their analyses identified 136 sexually dimorphic genes, with the majority being female-specific, largely attributable to the persistence of the X-zone in females. Moreover, bioinformatic analyses suggested enhanced Sonic hedgehog (Shh) signaling in males, whereas increased SPP1–integrin signaling was predicted in females. Spatial trajectory analysis using SpaTrack was applied to infer differentiation pathways and potentially define new ones. Finally, expression analyses using five different antibodies were conducted in an attempt to validate findings derived from the bioinformatic analyses.

We thank the reviewer for this careful summary of our work. Before addressing specific comments, we would like to highlight three major methodological improvements implemented in the revised manuscript that substantially strengthen the dataset and all downstream analyses.
We have re-analyzed all samples using Cellpose 3 (cyto3 model) for cell segmentation, replacing the default Space Ranger segmentation approach. The default Space Ranger pipeline performs nuclear detection followed by cytoplasmic expansion, which results in exclusion of cells whose cross-sections do not contain a nucleus in the tissue section plane — a particularly significant limitation in adrenal tissue sections where many steroidogenic cells are large and the nuclear plane may not be captured. Cellpose 3 performs whole-cell boundary detection directly from H&E morphology, recovering a substantially larger number of cells and providing more accurate cell boundary delineation. The resulting segmentation masks (.npy format) were supplied to Space Ranger via the --custom-segmentation parameter, enabling transcript assignment at true cell resolution. This re-analysis increased the total number of recovered cells to 203,069 cellsacross all eight samples (104,843 male, 98,226 female), substantially improving the depth and reliability of the atlas.
While centroid-based coordinates were used for cell-cell communication analysis (CellChat) and remain available through the interactive web atlas, full cell boundary segmentation masks are now utilized for spatial visualization within the Seurat framework (version 5), which natively supports cell boundary rendering. This allows visualization of gene expression at true single-cell resolution with accurate cell outlines rather than point-based representations, as reflected in the updated figures.
To ensure unbiased sex-comparative analyses, we identified a substantial imbalance in sequencing depth between male and female libraries (25.0 GB vs. 6.85 GB, ~3.65-fold difference). To correct for this technical bias, male FASTQ files were downsampled to match female sequencing depth prior to Space Ranger processing using seqtk (downsample fraction = 0.274, random seed = 42), and all analyses presented in this manuscript were performed on the depth-balanced dataset. The original undownsampled FASTQ files have been deposited in GEO alongside the processed data, allowing future users to take advantage of the additional sequencing depth for male-specific analyses where sex comparison is not the primary objective.
We also wish to draw the reviewer's attention to two important changes in the revised manuscript that alter the conclusions relative to the originally submitted version, and which explain apparent discrepancies with the reviewer's summary.
First, following re-analysis with the depth-balanced dataset and revised statistical thresholds, the total number of sexually dimorphic genes is now 1,128 at the whole-adrenal level (499 male-biased, 629

female-biased; adjusted $p < 0.05$, $|\log_2FC| > 0.5$), with 50 genes showing significant dimorphism specifically within the X/JMZ cluster (22 male-biased, 28 female-biased). These numbers differ from those reported in the original submission and reflect the improved analytical pipeline described above.
Second, regarding Sonic hedgehog signalling: following re-examination of the CellChat results on the reprocessed dataset, we were unable to confirm robust sex-differential Hedgehog/Shh pathway enrichment at the thresholds applied in the final analysis. Accordingly, we have removed references to male-enriched Shh signalling from the revised manuscript. The most female-enriched pathways in the revised analysis are SPP1. We apologize for any confusion this change may cause and have ensured that the revised manuscript clearly reflects these updated findings throughout.

Understanding the basis of sexual dimorphism in non-reproductive organs could shed light on sex-specific physiology and disease susceptibility. As such, this manuscript addresses a relevant and timely topic. However, the study is largely descriptive and many of the conclusions are drawn from bioinformatic analyses without functional validation. Consequently, the manuscript can be considered a resource rather than a basic research paper. As the vast majority of the findings are predictions, the claims are overly strong and should be toned down. Furthermore, I believe that achieving the level of impact appropriate for a Communications Biology publication would require additional experimental evidence.

We fully agree that the manuscript is primarily a resource paper providing a comprehensive spatial transcriptomic atlas, and we have revised the manuscript throughout to ensure that the language accurately reflects the descriptive and predictive nature of the findings. Specifically:
We have systematically toned down causal and mechanistic language across the Results, Discussion, and Conclusions sections. Phrases implying functional roles (e.g. "SPP1 maintains the X-zone", "bidirectional cell flow") have been replaced with appropriately qualified language (e.g. "SPP1 signalling is enriched in the female X-zone and may be associated with its maintenance", "spatial trajectory patterns consistent with bidirectional organization"). The word "predicted" has been added where CellChat-inferred interactions are discussed, and speculative interpretations are now explicitly framed as hypotheses requiring experimental validation.
We respectfully note, however, that high-quality spatial transcriptomic atlases of this resolution — combining near-single-cell Visium HD profiling, Cellpose-based cell segmentation, pseudobulk differential expression, cell-cell communication inference, spatial trajectory analysis, and immunohistochemical validation across eight biological replicates — represent a substantial methodological and biological contribution to the field, even in the absence of genetic loss-of-function experiments. The immunohistochemical validation of five sex-biased proteins (AEBP1, HMOX1, AKR1D1, NREP, CYP2F1) provides protein-level confirmation of key transcriptomic findings, and the publicly accessible interactive web atlas (https://adrenal-spatall-transcriptomic.shinyapps.io/adrenalsex/) provides the community with a reusable resource for hypothesis generation.
We believe that resource papers with rigorous methodology, multi-level validation, and open data access are consistent with the scope of Communications Biology, and we have framed the manuscript accordingly throughout the revision. The specific functional predictions generated here — including the role of SPP1-integrin signalling in X-zone maintenance and the bidirectional cortical trajectory model — are explicitly presented as testable hypotheses for future experimental studies, with concrete experimental approaches suggested in the Limitations and future directions section.

Specific points:
1. According to the Materials and Methods section, libraries were prepared from sections derived from four male and four female adrenals; however, apart from Fig. 1C/D, which show quite a bit of difference between sections (e.g. subcapsular populations), the authors present only merged datasets. It would be important to provide at least one supplementary figure showing U-Maps and cell distribution in individual samples to demonstrate consistency and reproducibility across biological replicates.

We thank the reviewer for this important point. We wish to draw attention to the fact that Figure 1C and 1D already display all individual tissue sections per sex simultaneously — all male sections are shown in panel C and all female sections in panel D — with cell population assignments overlaid on H&E-stained tissue for each section individually, allowing direct visual assessment of inter-replicate consistency in spatial zonation patterns.

We must, however, transparently acknowledge a technical limitation regarding sample composition that we have now clarified in the revised Methods and Limitations sections. To reduce sequencing costs, multiple adrenal glands were co-embedded in single paraffin blocks, and due to the geometry of co-embedding, individual sections captured tissue at different planes. In the male dataset, five sections were captured in total: four pass through the medullary compartment at varying depths, while one peripheral section captures only the subcapsular and CT capsule layers. In the female dataset, four sections were captured: two pass through the central plane including the medullary compartment and X-zone, while two are peripheral sections ending at the level of the inner ZF/X-zone boundary without medullary representation.

To ensure anatomically complete and balanced representation in sex-comparative analyses, pseudobulk differential expression and CellChat cell-cell communication analyses were performed exclusively on sections with complete adrenal cross-sections encompassing the medullary compartment and X-zone: four male and two female biological replicates. Peripheral sections lacking medullary representation were retained in the integrated atlas for cell type characterization, spatial visualization, and cortical zone analyses — where they increase cellular diversity and improve statistical power — but were excluded from sex-comparative DEG and communication analyses. This is now explicitly stated in the Methods section and acknowledged as a limitation in the Limitations and future directions section, where we note that future studies should section each adrenal gland individually to maximize biological replicate numbers.

The variability in subcapsular ZG representation visible between sections in Figures 1C/D reflects genuine sectioning plane variation inherent to the co-embedding strategy rather than biological inconsistency — and peripheral sections in fact provide enhanced resolution of the thin capsular progenitor niche.

In response to the reviewer's request, we have prepared Supplementary Figure S1 showing: (i) individual UMAP plots for each of the nine tissue sections colored by cluster identity, annotated to indicate sectioning plane (central vs. peripheral) and whether the section was included in DEG/CellChat analyses; and (ii) stacked bar plots of per-sample cluster proportions demonstrating consistency of cortical zone assignments across biological replicates. These data confirm that all major adrenocortical cell populations are consistently identified across biological replicates and that the fidelity of cluster assignments does not depend on any single section. Importantly, peripheral female sections F3 and F4, while excluded from pseudobulk DEG and CellChat analyses due to absence of the medullary compartment and X/JMZ zone, retain complete cortical coverage (CT capsule through inner ZF) and were included in the spatial trajectory analysis of the centripetal capsule-to-middle ZF axis, where they independently confirm the transcriptional gradient patterns observed in the central sections F1 and F2, supporting the robustness of the centripetal zonation findings across all four female biological replicates. The peripheral male section M5, which captures only the outermost subcapsular and CT capsule layers (n = 4,109 cells), was retained in the integrated atlas for cluster assignment and spatial visualization but excluded from pseudobulk DEG, CellChat, and trajectory analyses due to insufficient cortical depth. Collectively, these data demonstrate that the observed cortical zonation patterns are reproducible across biological replicates and are not driven by individual outlier sections.

2. Figure 2: Srd5a2 shows sexually dimorphic expression, with expression throughout the cortex in males but restricted to the X-zone in females. I assume that the images in Figure 2B represent graphical outputs of the Visium HD data. Are these scaled independently for each sex? In other words, are expression levels in the female X-zone directly comparable to those observed in the male cortex? This point should be clarified perhaps by using violin plots to provide actual read data. Ideally, this observation should be confirmed using RNA-scope analysis.

We thank the reviewer for this important methodological question. We confirm that all spatial expression maps in the manuscript use a shared color scale with identical expression boundaries applied simultaneously to both male and female sections, ensuring that color intensity reflects absolute normalized expression levels and that male and female images are directly comparable. This is now explicitly stated in the revised figure legend. Comparability between sexes is further strengthened by the sequencing depth balancing performed prior to analysis: as described in the revised Methods section, male FASTQ files were downsampled to match the sequencing depth of the female libraries (fraction = 0.274), eliminating the 3.65-fold depth imbalance present in the original data. Expression levels shown in all spatial maps and downstream analyses therefore reflect biological differences rather than technical depth artefacts.

We note that due to constraints on the number of spatial expression panels that could be accommodated in the main figures, *Srd5a2* spatial expression is not shown directly in the revised manuscript. However,

interactive spatial expression maps for *Srd5a2* and all other detected genes — including side-by-side male/female comparisons with a shared color scale — are freely accessible in our publicly available interactive web atlas at https://adrenal-spatall-transcriptomic.shinyapps.io/adrenalsex/. Importantly, the atlas includes a dedicated Gene Expression tab containing violin plots showing the distribution of normalized expression values per cell population per sex, which directly address the reviewer's request for quantitative read-level data.
Regarding orthogonal validation — we acknowledge that in situ hybridization confirmation would strengthen this observation. We have therefore added to the revised Discussion that RNAscope or Xenium in situ validation of key sexually dimorphic genes, including Srd5a2, Akr1c18, and Akr1d1, represents a priority for future studies. Xenium would be particularly well-suited in this context as it would simultaneously provide single-cell resolution spatial expression data and enable direct quantitative comparison with our Visium HD atlas within the same tissue architecture.

3. The reported higher expression of Shh in males is unexpected in particular when considering the higher recruitment of Gli1 positive capsular cells in females. Can this be confirmed using other samples (qPCR/ RNA-Scope)? Also, the sexually dimorphic expression of Itgav should be experimentally validated given that good antibodies are available.

We thank the reviewer for this insightful comment. We must, however, report that Shh was not detected as a significantly differentially expressed gene in our revised analysis. Following the methodological improvements implemented in this revision — specifically the application of Cellpose3 cell segmentation replacing the default Space Ranger nuclear segmentation, FASTQ-level sequencing depth balancing between male and female libraries, and pseudobulk differential expression analysis with edgeR using biological replicates — Shh did not reach statistical significance in any cluster-level or whole-adrenal comparison. We therefore wish to clarify that any reference to sexually dimorphic Shh expression in the original submission reflected a preliminary result that did not survive the more rigorous analytical framework applied in the revised manuscript. All mentions of Shh have been removed from the revised text, figures, and supplementary data. We apologize for any confusion this may have caused.

4. Spatial trajectory analysis: This type of analysis predicts ‘potential’ trajectories with the assumption that cells are differentiating. However, as the authors rightly point out in the discussion, transcriptomes may also change along signalling gradients without a true trajectory. As there are presently no true lineage tracing data for the X/JMZ zone the paragraph on bidirectional flow remains largely speculative and the title “Spatial trajectory inference reveals bidirectional cell flow’ needs to be changed.

We thank the reviewer for this important conceptual point and fully agree with the criticism. The term "cell flow" implies directed cellular movement supported by lineage tracing evidence, which we do not have. We have revised the section title to:
"Spatial trajectory inference reveals centripetal zonation and a centrifugal transcriptional axis in the adrenal cortex"
Throughout the revised text we have replaced language implying directed cell movement ("bidirectional flow", "cell migration", "bidirectional cell flow") with appropriately hedged terminology that reflects what the data actually demonstrate — namely, coordinated transcriptional gradients between spatially adjacent zones that are consistent with known cortical renewal biology but cannot be interpreted as direct evidence of cell lineage transitions in the absence of orthogonal lineage tracing data.
Specifically, the revised Discussion paragraph now explicitly states that: (i) spatial trajectory inference based on transcriptional similarity does not distinguish between true differentiation trajectories, signalling gradients, and zone-to-zone paracrine effects; (ii) the observed transcriptional continuum between the subcapsular ZG, ZF layers, and X/JMZ zone is consistent with the centripetal displacement model of adrenocortical renewal but requires Cre-based lineage tracing — for example using Nr5a1-Cre, Shh-Cre, or Gli1-CreERT2 driver lines — to establish true cellular ancestry; and (iii) the secondary centrifugal transcriptional axis between the JMZ/X region and inner ZF, which is particularly prominent in female samples with a complete X-zone cross-section (F1, F2), may reflect either active cell recruitment into the X-zone, a shared steroidogenic precursor identity, or spatial gene expression gradients maintained by paracrine signalling — none of which can be distinguished by transcriptomics alone. We believe these revisions appropriately temper the interpretive claims while preserving the descriptive value of the trajectory analysis as a hypothesis-generating tool for future lineage tracing studies.

5. The heatmaps of trajectory associated genes in Figure 7B appear to be different for male and female samples. This is surprising given that centripetal migration occurs in both sexes. Could the authors please show the heatmaps for male and female trajectory associated genes for both sexes? What does the 'trajectory' for the X/JMZ look like? Does it provide any meaningful clues regarding the transcriptional changes along the centrifugal axes? Are they convincing in all 4 female samples analysed?

We thank the reviewer for these insightful questions, which prompted us to extend and substantially improve the trajectory analysis.
Regarding sex differences in trajectory-associated genes (Figure 7B): The observation that heatmaps differ between male and female samples is expected and reflects genuine biological differences rather than a technical artefact. While the core steroidogenic differentiation program (*Cyp21a1*, *Star*, *Akr1b7*, *Cyp11b1*) is conserved across both sexes and appears at comparable pseudotime positions in all eight samples, the full complement of trajectory-associated genes differs between sexes, consistent with the pronounced transcriptional sexual dimorphism we document throughout the manuscript. To directly address this point, we have added Supplementary Figure S2A showing a cross-sex comparison of trajectory-associated genes along the capsule-to-middle ZF axis, with female (F1–F4) and male (M1–M4) samples displayed side by side. Genes consistently identified in ≥2 samples of each sex are ordered by peak pseudotime position in females, allowing direct visual comparison of expression timing and magnitude across sexes. This analysis confirms that core steroidogenic genes are active at comparable pseudotime positions in both sexes, while female-enriched early trajectory genes (*Fos*, *Gsn*, *Dcn*, *Dusp1*) and male-enriched extracellular matrix components show sex-specific enrichment at early pseudotime positions.
Regarding the JMZ/X-to-inner ZF centrifugal trajectory: We have added Figure 7C showing heatmaps of trajectory-associated genes along the JMZ/X-to-inner ZF centrifugal axis. This analysis was performed in all four male samples (M1–M4) and in the two female samples (F1, F2) that contain a complete cross-section through the X-zone. The remaining female samples (F3, F4) lack sufficient representation of the JMZ/X region for this trajectory to be reliably reconstructed, as these sections captured peripheral cortical planes lacking the corticomedullary compartment. The centrifugal trajectory analysis does provide meaningful biological insights: female samples show prominent expression of established X-zone markers (*Akr1c18*, *Akr1d1*, *Cyp11a1*, *Aldh1a1*) along this axis, consistent with the transcriptionally active X-zone in pre-pubertal females, while male samples show markedly reduced expression of these markers, reflecting the regressed or absent X-zone. A cross-sex comparison of the centrifugal trajectory is provided in Supplementary Figure S2B. We note that these transcriptional gradients along the JMZ/X-to-inner ZF axis should be interpreted as reflecting zone-specific transcriptional states rather than confirmed directional cell migration, consistent with our revised interpretation throughout the manuscript.
Regarding convincingness across all four female samples: We acknowledge that the centrifugal trajectory analysis could only be performed in two of four female samples (F1 and F2) due to the tissue sectioning plane. This is explicitly stated as a limitation in the revised Results section and in the Limitations paragraph of the Discussion. The consistency of X-zone marker expression between F1 and F2, combined with the spatial transcriptomic and immunohistochemical data from all four female sections demonstrating X-zone-specific expression of *Akr1c18*, *Akr1d1*, and *Pik3c2g*, supports the biological robustness of the observed centrifugal transcriptional axis despite the reduced sample number available for formal trajectory reconstruction.

6. Osteopontin as a mechanism to maintain the X-zone: Osteopontin expression in the female X-zone has been previously reported. While SpaTrack predicts interactions with integrins, the presented data do not demonstrate that X-zone maintenance is mediated through this pathway (as claimed in the discussion). Ideally, analysis of adrenal tissue from Spp1 knockout mice should be analyzed (these ko mice survive and are fertile) to provide functional data and to support this hypothesis. If such experiments are not feasible, the claim of osteopontin signaling as a mechanism of maintaining the X-Zone in females should be rewritten and shortened to reflect the speculative nature of the findings.

The criticism is fully justified and we agree that the original Discussion overstated the functional significance of SPP1 signalling in X-zone maintenance. The revised Discussion section now accurately reflects the correlative and hypothesis-generating nature of this observation.
The data presented demonstrate: (i) significantly higher *Spp1* expression in the female X/JMZ cluster compared to males; (ii) spatial co-localization of *Spp1* ligand with integrin receptor subunits

(*Itgav*, *Itgb1*, *Itgb5*) in the female X-zone; and (iii) computational prediction of SPP1-integrin interactions by CellChat. These observations are consistent with active SPP1-mediated intercellular communication in the female X-zone microenvironment but do not demonstrate causality or establish SPP1 as a mechanism of X-zone maintenance.
The subheading of the relevant Discussion section has been changed from *"Osteopontin (SPP1) signalling as a female-specific X-zone maintenance mechanism"* to *"Osteopontin (SPP1) signalling is enriched in the female X-zone and represents a candidate paracrine communication pathway"*. The concluding sentences of the paragraph have been rewritten to remove all mechanistic claims and replace them with explicitly hedged language, acknowledging that whether SPP1-integrin signalling is required for X-zone survival, regulation of *Akr1c18* expression, or resistance to androgen-mediated involution cannot be determined from transcriptomic data alone. The revised text proposes *Spp1* knockout mice — which are viable and fertile — as the appropriate experimental model to test this hypothesis, and frames SPP1-integrin signalling explicitly as a hypothesis-generating observation and priority target for future functional investigation rather than an established mechanism.

7. Figure 8: HMOX1 and AEBP1 immunostaining shows strong signals in the medulla, whereas these cells appear negative in the spatial transcriptomic analysis. This discrepancy raises concerns regarding antibody specificity, which should be addressed.
The Discussion section is rather long and contains several claims that lack sufficient experimental support:

We thank the Reviewer for this careful observation. We fully acknowledge the discrepancy between the medullary immunoreactivity observed for HMOX1 and AEBP1 and the absence of a corresponding signal in the spatial transcriptomic data, and agree that this requires explicit discussion rather than a passing remark.
We have made two targeted changes to address this:
1. Results section (IHC description, after the sentence describing HMOX1 sex differences in the cortex), and
2. Discussion section: "Protein-level validation confirms transcriptomic sex differences in the X-zone" (replacing the existing brief remark on this issue). We believe these additions adequately contextualise the observed discrepancy, acknowledge the limitation transparently, and provide a scientifically grounded framework for its interpretation without overstating its impact on the principal conclusions of the study, which rest on concordant transcriptomic and IHC data for the X-zone markers AKR1D1, NREP, and CYP2F1.

8. The statement that “this study presents the first comprehensive high-resolution spatial transcriptomic … revealing profound sexual dimorphism that is spatially restricted to inner cortical zones” is very strong. Doesn't the authors’ own data suggest that Hmox1 is sexually dimorphic throughout the cortex?

We thank the Reviewer for this astute observation. The Reviewer is entirely correct — our own spatial data show that *Hmox1* displays diffuse male-biased expression extending across multiple cortical zones rather than being confined to the inner cortex, and the original phrasing was therefore inconsistently strong relative to our findings.
We have revised the relevant passages throughout the manuscript to reflect a more accurate characterisation. Specifically, the phrase "spatially restricted to inner cortical zones" has been replaced consistently across the Abstract (two instances), the opening paragraph of the Discussion, the section "Spatial restriction of sexual dimorphism to inner cortical zones", and the Conclusions. In all locations, the revised wording acknowledges that while the most extreme sex differences, including the X-zone molecular signature, are concentrated in the inner cortex and corticomedullary boundary, selected male-biased genes such as *Hmox1* exhibit diffuse expression across multiple cortical zones. The unifying formulation adopted throughout is that sexual dimorphism in the mouse adrenal cortex is *zonal in emphasis rather than absolute in anatomical confinement*, which we believe accurately reflects our data while preserving the central conclusion that the inner cortex is the dominant locus of adrenal sexual dimorphism in the sexually mature mouse.

9. The X-zone–specific expression of Akr1c18 (and its female specificity) was reported several decades ago. This section of the Discussion should therefore be substantially shortened.

We thank the Reviewer for this valid criticism. We agree that the X-zone-specific and female-predominant expression of *Akr1c18*/20α-HSD is well established in the literature and did not require the level of contextualisation provided in the original manuscript. We have substantially shortened this section, removing the introductory akapit describing X-zone biology (involution dynamics, homology to the human fetal zone, species specificity) and the akapit detailing the biochemistry of 20α-HSD and the Hershkovitz model, as these recapitulate knowledge that is decades old and widely available in cited reviews.
The revised section retains only content that represents a genuine new contribution of the present study: the quantification of *Akr1c18* female specificity at single-cell spatial resolution ($\log_2$FC = −16.28), a cautionary note regarding its use as a universal marker in light of its reported expression in the definitive lineage in certain knockout models, and the identification of additional female-enriched X-zone markers (*Akr1d1*, *Pik3c2g*, *Cd1d1*) that extend our understanding of X-zone steroidogenic capacity beyond progesterone catabolism. The section has been reduced from four paragraphs to two.

10. Paragraph: “Sex differences in intercellular communication networks: complexity versus pathway specificity.”
Bioinformatic predictions of pathway activity are inherently challenging and should generally be viewed as a basis for future experimentation. Gene expression alone does not necessarily imply pathway activation or biological relevance in this context. Statements such as “the X-zone serves as a centralized signalling hub (as evidenced by SPP1)” are therefore not sufficiently supported and should be revised.

We thank the Reviewer for this important methodological point, which we fully accept. We agree that CellChat-based inference of intercellular communication represents a computational prediction grounded in ligand-receptor co-expression rather than direct experimental evidence of pathway activation, and that the original manuscript did not sufficiently reflect this limitation in its language.
We are pleased to note that the concerns raised by the Reviewer have already been addressed during the current revision of the manuscript. Specifically:
The statement *"the X-zone serves as a centralized signalling hub (as evidenced by SPP1)"* has been removed from the Discussion. In the SPP1 section, the relevant passage has been revised to reflect the correlative and hypothesis-generating nature of these findings. The revised text now explicitly states that SPP1 signalling *"represents a candidate paracrine communication network"*, that *"these observations are correlative and do not establish a functional role for SPP1 in X-zone maintenance"*, and that *"whether SPP1-integrin signalling is required for X-zone survival... cannot be determined from transcriptomic data alone"*. SPP1-integrin signalling is now framed explicitly as *"a hypothesis-generating observation and a priority target for future functional investigation"*.
We believe these revisions bring the Discussion into alignment with the Reviewer's expectation that bioinformatic predictions of pathway activity be presented as a basis for future experimentation rather than as evidence of biological function.

Minor points
9. First paragraph: The statement “We obtained ~104,000 high-quality single-cell transcriptomes” is misleading. These data represent inferred single cells derived from spatial transcriptomics, which does not allow unambiguous single-cell resolution. This should be clarified.

The original wording was imprecise and has been corrected throughout the revised manuscript. Visium HD generates spatially resolved transcriptomes at 2×2 μm bin resolution, and while Cellpose3-based cell segmentation assigns transcript-containing bins to morphologically defined cell boundaries — substantially improving cellular resolution compared to standard spot-based approaches — this does not constitute true single-cell resolution in the sense of microfluidics-based single-cell RNA sequencing.

Cell boundaries inferred from H&E morphology may encompass portions of adjacent cells, and transcript assignment to segmented units remains probabilistic rather than unambiguous.
The revised manuscript therefore replaces *"single-cell transcriptomes"* with *"cell-level transcriptomes derived from Cellpose3-based segmentation of Visium HD data"*

10. Experimental setup: The authors used 7-week-old mice, which should be considered adolescent rather than fully adult. This limitation should be explicitly acknowledged.

We thank the Reviewer for raising this point. We are pleased to confirm that this limitation has already been explicitly acknowledged in the revised manuscript. The Limitations section now reads:
"The age of 7 weeks should be considered adolescent rather than fully adult, and sex-specific transcriptional patterns may differ in older animals; future studies should include multiple age groups to capture the full developmental trajectory of adrenal sexual dimorphism."
This addition was introduced during the current revision in the context of a broader limitation regarding the single-timepoint nature of the study, and directly addresses the Reviewer's concern.

11. Discussion, page 17: The sentence “we performed immunohistochemical validation of selected markers (Figure 9)” should refer to Figure 8, not Figure 9.
Corrected

Reviewer #3 (Remarks to the Author):

The adrenal gland is recognized as one of the most sexually dimorphic organs. Understanding sex-dependent mechanisms in adrenal gland function is critical, as many adrenal diseases occur more frequently in females. Despite differences in structure and steroid production between humans and rodents, they share many similarities, and transgenic mouse models have proven useful for deciphering molecular mechanisms in adrenal development and pathology.
In the present manuscript entitled ‘Single-cell spatial transcriptomics reveals sex-dependent gene expression and intercellular signaling in mouse adrenal cortex’, Blatkiewicz and colleagues employ Visium HD spatial transcriptomics to profile transcriptomes from 7-week-old male and female adrenals and explore sex-dependent mechanisms in the adrenal gland. The authors present a valuable accessible resource, which is appreciated. However, some aspects require clarification, some figures would benefit from reorganization, and some statements need to be softened. Nevertheless, once revised, this manuscript is expected to provide critical information for the scientific community and has the potential to be well cited. Below are our detailed comments.
Major comments:
1) Line 37: The claim that ‘females engage SPP1-integrin pathways for X-zone maintenance’ requires experimental validation. Such a conclusion should be rephrased (i.e., the data suggests...) unless functional experiments are provided (for example, Spp1 KO model).

We thank the Reviewer for this valid criticism. We fully agree that the original phrasing overstated the functional significance of the SPP1-integrin findings, which are based solely on transcriptomic and spatial co-expression data and have not been experimentally validated.
We have reviewed all instances of this claim throughout the manuscript. The most direct instance — *"females engage SPP1-integrin pathways for X-zone maintenance"* — was already removed from the Abstract during the current revision. The remaining instance in the Conclusions has now been revised from *"pathway-specific mechanisms (SPP1-integrin signalling) associated with X-zone maintenance"* to *"pathway-specific mechanisms, with SPP1-integrin signalling enriched in the female X-zone microenvironment and representing a candidate paracrine communication pathway warranting functional investigation."*
These revisions are consistent with the language adopted throughout the SPP1 Discussion section, where SPP1-integrin signalling is explicitly described as correlative, hypothesis-generating, and in need of functional validation using models such as *Spp1* knockout mice.

2) Akr1c18 as a ‘definitive marker’ (Line 35) or ‘gold-standard’ (Line 498) is debatable. Indeed, mouse models such as Prkar1a KO and Hhex KO, with increased Akr1c18 expression in males despite the absence of X-zone and Pik3c2g expression, suggest that Akr1c18 can be expressed in cells of the definitive lineage. Perhaps the authors could soften the conclusion or focus on Pik3c2g, which seems to be a consistent X-zone marker across studies and mouse models.

We thank the Reviewer for this important point, which we fully accept. The Reviewer is correct that *Akr1c18* expression in *Prkar1a* and *Hhex* knockout models — where it appears in cells of the definitive lineage despite the absence of *Pik3c2g*expression — argues against its use as a universal gold-standard marker.
The term "gold-standard marker" has already been removed from the Discussion during the current revision, replaced by a passage explicitly cautioning against this interpretation and proposing combined use of *Akr1c18* and *Pik3c2g* for more robust X-zone identification. The remaining instance of "definitive marker" in the Abstract has now been revised to "primary X-zone marker", with the addition of a note that its use in combination with *Pik3c2g* provides more robust zone identification. This language is now consistent throughout the manuscript and directly reflects the Reviewer's suggestion to focus on *Pik3c2g* as the more reliable marker across mouse models.

3) Line 38, I would recommend choosing a different word than ‘establish’ since the X-zone capacity to metabolize progesterone has been demonstrated since 2007 at least (Hershkovitz et al., cited line 491).

We thank the Reviewer for this precise observation. The Reviewer is entirely correct — the progesterone-catabolizing function of the X-zone was demonstrated by Hershkovitz et al. in 2007, and the use of "establishing" implies a novelty of discovery that is not warranted. We have replaced "establishing" with "confirming" in all relevant instances throughout the manuscript, including the Abstract, the Results section summary of IHC validation, and the Discussion. This change accurately positions our spatial transcriptomic data as high-resolution corroboration of previously described X-zone biology rather than its original demonstration.

4) Line 65, the statement that the ‘molecular basis (of adrenal sexual dimorphism) remains poorly understood’ is not accurate. There has been considerable characterization over the last decade, particularly implicating the androgen receptor (AR), its target gene Hhex, Wnt signaling, senescence, immune response etc…. Please revise to acknowledge and provide the reader with the most recent existing publications. Among few examples: Dolfi et al., 2022. Unravelling the sex-specific diversity and functions of adrenal gland macrophages. Wang et al., 2025. The Transcriptome Trajectory Reveals Sex- and Age-Dependent Changes in the Mouse Adrenal Gland, Takahashi et al., 2024, Development of sexual dimorphism of skeletal muscles through the adrenal cortex, caused by androgen-induced global gene suppression., Dumontet et al. 2026, The transcription factor HHEX maintains glucocorticoid levels and protects adrenals from androgen-induced lipid depletion. Grabek and al., 2019 demonstrated that females employ an additional stem and/or progenitor compartment. This information is lacking in the introduction, while it could perhaps support the analysis the authors performed in Figure 5D.

We thank the Reviewer for this substantive criticism and for providing specific references. The Reviewer is entirely correct that the original phrasing failed to acknowledge the considerable progress made over the past decade in characterising the molecular mechanisms of adrenal sexual dimorphism.
The phrase "remains poorly understood" was already revised during the current revision to "remains incompletely characterised at single-cell spatial resolution", which better defines the specific gap addressed by our study. However, we agree that the Introduction required a more explicit acknowledgement of the existing mechanistic landscape.
We have therefore added a dedicated paragraph to the Introduction recognising: androgen-induced global suppression of zona fasciculata gene transcription mediated by NR5A1 (Takahashi et al., 2024); the role of *Hhex* in maintaining glucocorticoid levels and protecting the adrenal cortex from androgen-induced lipid depletion (Dumontet et al., 2026); sex-specific adrenal immune cell composition (Dolfi et al., 2022); age-dependent transcriptomic trajectories showing progressive divergence of male and female adrenal transcriptomes from postnatal day 28 (Wang et al., 2025); and the female-specific

capsular Gli1-positive progenitor compartment (Grabek et al., 2019). The paragraph concludes by defining the specific gap that our study addresses — the spatial organisation of sex-biased gene expression and intercellular communication at near-single-cell resolution — clearly distinguishing our contribution from existing bulk and single-cell studies.
Regarding Grabek et al. (2019): we have additionally added a brief note in the Results section linking the female-specific progenitor compartment to the sex-specific signalling patterns observed in Figure 5D, where the subcapsular zona glomerulosa emerges as a dominant communication hub with distinct pathway engagement between sexes, potentially reflecting the differential progenitor niche architecture described by Grabek and colleagues.

5) Perhaps the authors could clarify the knowledge gap they address in the present manuscript. Is it about the X-zone, the cell-cell communication, etc..? I recommend highlighting more the accompanying web app for data visualization, both in the abstract and the introduction.

We thank the Reviewer for this constructive suggestion. We agree that the original manuscript did not sufficiently define the multiple knowledge gaps addressed by the study, and that the interactive web resource was undersold.
We have made two targeted changes. First, the knowledge gap statement in the Introduction has been revised to explicitly enumerate the three interconnected gaps addressed by this study: the spatial organisation of sex-biased gene expression at near-single-cell resolution, the comprehensive molecular characterisation of the X-zone, and the architecture of sex-dependent intercellular communication networks in the adrenal cortex. Second, the URL of the interactive web application (https://adrenal-spatall-transcriptomic.shinyapps.io/adrenalsex/) has been added to both the Abstract and the Introduction, where it is presented as an open resource enabling exploration of the spatial atlas. It was previously mentioned only in the Results and Conclusions sections.

6) Figure 5 and 6 could be organized together for better continuity, as well as 3 & 4. Figure 8 should accompany figure 4, the current organization is confusing.

We thank the Reviewer for this suggestion regarding figure organisation. We note that the Reviewer's comments refer to the figure numbering in the originally submitted version. During the current revision, we have already reorganised the figure sequence to improve continuity: the spatial maps of sex-biased gene expression (originally Figure 4) and the differential gene expression analysis across clusters (originally Figure 3) have been reordered so that they now appear in direct succession, and the cell-cell communication figures (originally Figures 5 and 6) have been consolidated into a single section.
Regarding the suggestion to place the IHC validation figure (originally Figure 8) adjacent to the spatial expression maps: we have moved the IHC figure forward in the manuscript so that it follows more directly from the spatial gene expression data it validates. We acknowledge, however, that a complete structural reorganisation is constrained by the logical flow of the narrative, which proceeds from atlas construction → sex-biased expression → trajectory analysis → cell-cell communication → protein validation. We believe the revised order represents the best balance between the Reviewer's suggestion and narrative coherence, and we have ensured that cross-references between figures are clearly indicated in the text to guide the reader.

7) Figure 3H and Figure 4. The increased expression of Akr1c18 in the medulla is likely due to steroidogenic cells localized among medulla cells. Perhaps the authors could add Sf1 to their panel to assess this. If accurate, could the authors comment on the spatial method and its limitations in regard to misidentifying/mis-clustering small pockets of cells belonging to a different lineage than the tissue in which they are found?

We thank the Reviewer for this insightful observation. We agree that the scattered *Akr1c18*-positive signal observed within the medullary compartment in female spatial maps most likely reflects the well-documented presence of steroidogenic cortical cells interdigitating within the chromaffin cell mass, a phenomenon described in the adrenal histology literature, rather than true *Akr1c18* expression in

chromaffin cells. The text already noted these as *"scattered cortical cells embedded within the medullary compartment"*, but did not sufficiently contextualise this observation.
We have made two additions to address this. First, in the Results section, we have added a sentence explicitly attributing the medullary *Akr1c18* signal to interdigitating steroidogenic cells identifiable by Nr5a1 (Sf1) expression, and acknowledging that Nr5a1 immunostaining was not included in the current validation panel, representing a limitation that future studies should address.
Second, we have added a dedicated point to the Limitations section discussing the general challenge of mis-clustering small pockets of cells belonging to a lineage different from the surrounding tissue in Cellpose-based spatial transcriptomic segmentation. In the adrenal gland specifically, cortical steroidogenic cells interdigitating within the medulla may be assigned to the medulla cluster based on spatial proximity rather than true lineage identity. We propose that incorporation of lineage-specific markers, *Nr5a1* for steroidogenic cells and Th or Pnmt for chromaffin cells, into future spatial panels would enable more precise cell identity assignment at the corticomedullary boundary.
Regarding the suggestion to add Sf1 immunostaining to the current panel: while we appreciate this recommendation, performing additional immunohistochemical experiments at this stage of revision is not feasible. We have therefore chosen to address this point transparently as a limitation rather than claim to have resolved it experimentally.

8) In the results section, it would be valuable to briefly introduce the functions of genes listed. For example, Susd3 and Ddx3y are not mentioned in the text, yet the authors show in Figure 4 that they are sexually dimorphic in the zG and outer zF. I suggest these two genes, Mt1 and mt-Nd4 be added to the panel of spatial transcriptomic maps as well. Aebp1 function is also not mentioned, while the authors put efforts into validating this gene by immunohistochemistry.

We thank the Reviewer for this valuable suggestion. We agree that the Results section would benefit from brief functional annotations of the genes discussed, particularly those validated by immunohistochemistry or highlighted in figures.
We have made the following additions. First, a brief functional description of *Susd3* (complement regulatory protein implicated in cell adhesion and immune modulation) and *Ddx3y* (Y-chromosome-linked RNA helicase involved in transcriptional regulation, previously identified as a consistent male-specific adrenal marker by Wang et al., 2025) has been added to the Results section, together with a note on their sexually dimorphic spatial distribution in the zona glomerulosa and outer zona fasciculata. Second, a functional annotation of AEBP1 (a transcriptional repressor with roles in adipogenesis, inflammation, and extracellular matrix remodelling) has been added to the IHC results paragraph, contextualising its male-biased adrenal expression.
Regarding the suggestion to add *Mt1* and *mt-Nd4* to the spatial expression panel: we appreciate this recommendation, and acknowledge that the spatial distribution of these genes would be of interest. However, the figure panel already contains twenty genes selected to represent the top sex-biased markers across all major adrenal cell populations, and further expansion would compromise figure readability and exceed the space constraints of the manuscript. Importantly, spatial expression data for *Mt1*, *mt-Nd4*, and all other genes detected in this dataset are fully accessible through the accompanying interactive web application (https://adrenal-spatall-transcriptomic.shinyapps.io/adrenalsex/), which we have highlighted more prominently in both the Abstract and Introduction in response to comment 5. We therefore respectfully suggest that the web resource represents the most appropriate venue for exploration of individual gene spatial patterns beyond the curated panel presented in the figures.

9) Line 408: The authors describe AKR1D1 expression domain as a 'sharply demarcated band at the corticomedullary boundary in females corresponding precisely to the X-zone', but the spatial maps presented in Figure 4 showed a clear wider expression pattern than Akr1c18. Are the authors suggesting that mRNA and protein expression are incompletely overlapping?

We thank the Reviewer for this precise and important observation. The Reviewer is correct that the original description of AKR1D1 protein localisation as *"corresponding precisely to the X-zone"* was inconsistent with the spatial transcriptomic data, which already noted in the Results that *Akr1d1* mRNA

exhibits *"broader, more diffuse expression extending throughout the inner cortical layers"* compared to the sharply demarcated *Akr1c18* pattern. This internal inconsistency has now been resolved.
The IHC Results section has been revised to explicitly acknowledge that the AKR1D1 protein distribution is broader than the *Akr1c18* mRNA domain, extending into inner cortical layers beyond the strict X-zone boundary — consistent with the spatial transcriptomic data. We further note that this incomplete spatial overlap between *Akr1d1* mRNA and AKR1D1 protein is consistent with post-translational regulatory mechanisms or differential protein stability, and indicate that while *Akr1d1* is strongly female-enriched, its expression domain is wider than that of *Akr1c18*. A corresponding note has been added to the Discussion section on protein-level validation. We therefore confirm that yes — mRNA and protein expression for AKR1D1 are incompletely overlapping, and the revised text now addresses this directly rather than implying full concordance.

10) In regards of Per1 pattern in the trajectory analysis, were all the samples collected at the same timepoint? If yes, provide this information in the methods section and regard the strong circadian rhythmicity of Per1 expression.

We thank the Reviewer for raising this important point. All eight adrenal samples were collected at the same time of day, minimising circadian variation as a confounding factor. We have added this information explicitly to the Methods section.
Regarding *Per1*: we note that the passage in which *Per1* appeared as an early trajectory gene in the female-specific trajectory analysis was part of a section that has been revised and substantially shortened during the current revision. In the updated manuscript *Per1* no longer features prominently in the trajectory discussion. We nonetheless acknowledge the Reviewer's point that *Per1*, as a core circadian clock gene with strong rhythmic expression, is inherently difficult to interpret in the context of spatial trajectory inference from a single temporal snapshot, and we have added a brief cautionary note to this effect in the trajectory Results section for completeness.
We would also note that the use of FFPE tissue, while advantageous for spatial transcriptomics, introduces an additional layer of complexity in interpreting circadian gene expression, as fixation captures a single transcriptional state. Future studies incorporating time-resolved spatial transcriptomics across multiple zeitgeber timepoints would be required to disentangle spatial gradients from circadian oscillations for genes such as *Per1*.

11) CYP2F1 data are confusing. I suggest removing it or explaining the rationale for the analysis and the conclusion it supports in greater detail. Is this piece of data related to CYP2F2 expression in the inner zF by any means?

We thank the Reviewer for this observation and appreciate the opportunity to clarify. CYP2F1 was included in the IHC panel as a protein-level surrogate for *Cyp2f2*, a female-enriched gene identified in the spatial transcriptomic analysis as broadly expressed in the inner cortical layers and the X-zone. CYP2F1 and CYP2F2 are closely related members of the cytochrome P450 2F subfamily sharing considerable sequence homology, and the available commercial antibody was selected based on predicted cross-reactivity with mouse CYP2F2.
The finding that CYP2F1 immunostaining showed consistently low expression in both sexes, with only marginal X-zone enrichment in females, is itself informative: it suggests that despite robust *Cyp2f2* mRNA detection in the spatial transcriptomic data, protein-level expression may be subject to post-translational regulation, rapid protein turnover, or that the antibody cross-reactivity with mouse CYP2F2 is insufficient for reliable detection under the conditions employed. This observation is therefore retained in the manuscript as a cautionary example of the limitations of mRNA-to-protein inference, and the Results section has been revised to make this interpretation explicit rather than leaving the data uncommented. We acknowledge that the original text did not provide sufficient context for this result, and we have added a clarifying sentence explaining the rationale for including CYP2F1 and the biological interpretation of the negative finding.

12) Line 417: 'These findings confirm that sexual dimorphism in the mouse adrenal gland is spatially restricted to the corticomedullary boundary'. This statement contradicts Figure 4: Hhex, Acsbg1, Mgarp,

and Jun are clearly sexually dimorphic and not restricted to the corticomedullary boundary. I suggest revising the conclusions or clarifying their meaning.

We thank the Reviewer for identifying this inconsistency. The Reviewer is entirely correct, genes such as *Hhex*, *Acsbg1*, *Mgarp*, and *Jun* display male-biased expression extending across multiple cortical zones, and the original statement was therefore overly restrictive and inconsistent with our own data.
This issue is related to the broader revision made in response to comment 8 of Reviewer 1, where the phrase "spatially restricted to inner cortical zones" was replaced throughout the manuscript with language reflecting that sexual dimorphism is *zonal in emphasis rather than absolute in anatomical confinement*. The IHC summary sentence has now been brought into alignment with this revised framing: it now states that the most pronounced sexual dimorphism at the protein level is *concentrated* at the corticomedullary boundary, while explicitly acknowledging that genes such as *Hhex*, *Mgarp*, *Jun*, and *Acsbg1* exhibit male-biased expression across broader cortical regions. This revision ensures consistency between the IHC Results, the spatial transcriptomic data, and the Discussion throughout the manuscript.

13) From lines 337 to 384: The spatial trajectory analysis is interesting, and the authors suggest bidirectional flow. However, the second trajectory originates from the X/JMX, but the GAM analysis seems to be missing for this trajectory.

We thank the Reviewer for this careful observation. The Reviewer is correct that the original manuscript lacked GAM analysis for the secondary centrifugal trajectory originating from the X/JMZ region. This was an oversight that has now been addressed.
GAM analysis has been performed along the JMZ/X-to-inner ZF axis for all male samples and for female samples with complete corticomedullary representation (F1 and F2). The resulting trajectory-associated genes are presented as a heatmap in an additional panel added to the trajectory figure, and the Results section now includes a description of the gene expression modules identified along this centrifugal axis. The identification of established X-zone markers among the top GAM-associated genes supports the biological validity of the inferred centrifugal trajectory and reinforces the interpretation that the X/JMZ compartment maintains a transcriptionally distinct and spatially autonomous programme.
We note that the legend for the trajectory figure already contained a reference to this panel (described as panel C, showing heatmaps of trajectory-associated genes along the JMZ/X-to-inner ZF axis) as part of the current revision; the Results text has now been updated to provide the corresponding description of the genes identified, ensuring full consistency between figures and text.

Minor comments:
1) Line 35, statistical details in the abstract are unnecessary and could be restricted to the results and method sections.
2) Add the age in the results section and the legend of the figure (n=4 males, 4 females, 7 week-old).
3) Line 314: Figure 6A and 6B are flipped in the result section.
4) Scales are missing from all histology pictures.

Typos:
1) Line 100: in the introduction: ‘spatial restriction
2) Line 102: Specialized in progesterone catabolism instead of specialized that progesterone catabolism

We thank the Reviewer for these detailed observations. All corrections suggested under the minor comments and typos have been implemented.

Reviewer #4 (Remarks to the Author):

I co-reviewed this manuscript with one of the reviewers who provided the listed reports. This is part of the Communications Biology initiative to facilitate training in peer review and to provide appropriate recognition for Early Career Researchers who co-review manuscripts.

We thank Reviewer #4 for participating in the peer review process of this manuscript and for their contribution to the evaluation alongside the primary reviewer. We appreciate the Communications Biology initiative to support Early Career Researchers in developing peer review expertise, and we hope that our detailed responses to the reviewers' comments and the revised manuscript will meet their expectations.

Reviewers' comments:

Reviewer #1 (Remarks to the Author):

This reviewer has no additional comments. This dataset will be a valuable resource for the adrenal research community.

Response to Reviewer #1

We sincerely thank Reviewer 1 for their positive assessment and for acknowledging the potential of this dataset as a resource for the adrenal research community. We appreciate the time and effort dedicated to reviewing our manuscript across both rounds of review.

Reviewer #2 (Remarks to the Author):

The authors have taken most of my comments into account and the more careful wording is now acceptable. I appreciate the new analysis using data that were adjusted for sequencing depth, and the use of the Cellpose 3 algorithm. Additional experiments to support their hypothesis on the osteopontin/SPP1 axis would have been nice, but perhaps this is beyond the scope of this paper.

Minor point:
Please double check the official nomenclature (e.g. I spotted a couple of places were knockouts were not labelled in italic):
Mouse protein: SHH (Capital, straight)
Mouse gene, RNA, and knockouts: *Shh* (Only first letter capital, gene name in italic)

Response to Reviewer #2

We sincerely thank Reviewer 2 for the thorough and constructive review across both rounds, and for acknowledging the improvements introduced in the revised manuscript. We have checked the manuscript for official mouse nomenclature and corrected gene, RNA, knockout, and protein formatting throughout. Mouse genes and RNAs are now italicized with only the first letter capitalized, e.g. *Shh*, *Spp1*, *Cyp2f2*, whereas protein names are presented in upright capitalized form, e.g. SHH, SPP1, CYP2F2.

Reviewer #3 (Remarks to the Author):

I read the answers provided by the authors to the reviewers comments and my major concerns are:

The functional validation requested by the reviewers have been declined by the authors, including validation of SPP1 pathway at the protein level which is now the main point of the current manuscript, after removal of Shh expression data. Overall, the current manuscript is an important technical innovation for the field, but the results presented are not novel or strongly supported by functional analysis/orthogonal approaches.

It appears that the dataset has been drastically reanalyzed. According to the authors, this was in part to correct a technical bias. As a result, conclusions of the manuscript have been altered, including the sexually dimorphic expression of Shh. According to the authors, this 'reflected a preliminary result that did not survive more rigorous analytical framework'. Another concern is the use of antibodies with cross-reactivity that might be 'insufficient for reliable detection under the conditions employed' according to the authors. These statements alone raise significant concern regarding the scientific rigor. The staining for CYP2F1 has no value in the current manuscript and do not replace staining for Cyp2f2, antibodies suitable for IHC are available and already been published (Lyu et al 2020, endocrinology)

Additional comments: potential panel duplication between figure 2 and figure 3 (abcb1b, akr1b7).

Response to Reviewer #3

We thank the reviewer for the thorough and critical assessment, which has helped us substantially improve the clarity and scientific rigor of the manuscript. We address each concern in turn.

We thank the reviewer for raising this point and take it seriously. We agree that functional validation of candidate signalling pathways, including SPP1-associated signalling, would be required to establish causal mechanistic roles. We wish to clarify, however, that the primary objective of the present study is to provide a rigorously curated spatial transcriptomic resource and to identify candidate signalling programs associated with sex-specific epithelial states, rather than to establish functional roles for individual pathways.

We would like to respectfully highlight that this study offers several contributions that are genuinely novel: (i) the first near-single-cell-resolution spatial transcriptomic atlas of the sexually dimorphic mouse adrenal gland (~203,000 Cellpose 3-segmented transcriptomes from eight biological replicates); (ii) the first systematic spatial mapping of sex-biased intercellular communication networks in the adrenal cortex; (iii) identification of a secondary centrifugal transcriptional axis emanating from the JMZ/X compartment; and (iv) an open interactive web atlas enabling community-wide data exploration. These contributions represent advances that bulk RNA-seq and microarray approaches cannot provide, precisely because spatial resolution reveals the zonal restriction of sex-biased gene expression. Regarding the SPP1 pathway specifically, computational inference of ligand-receptor signalling from spatial transcriptomic data without concurrent functional validation is now standard practice in atlasing studies across multiple tissues and organs. The CellChat framework is designed for hypothesis generation, and its authors explicitly frame inferred interactions as predictions requiring future experimental follow-up, which is precisely the framing we have adopted in the revised manuscript. In response to the reviewer's concerns, we have substantially toned down the interpretation of all SPP1 findings. We now consistently refer to SPP1-integrin signalling as an inferred, hypothesis-generating pathway association, and we no longer present SPP1 as a validated mechanistic axis or as being required for X-zone maintenance.

To provide orthogonal support without overstating mechanistic conclusions, we note that the biological plausibility of SPP1 enrichment in the female X-zone microenvironment is supported by multiple independent published datasets. Lyu et al.

(Endocrinology, 2020, ref. 55) demonstrated by immunohistochemistry that SPP1 protein localizes to the mouse adrenal inner cortex partially colocalizing with the X-zone marker 20α-HSD, and identified SPP1 as one of the top sexually dimorphic genes responsive to thyroid hormone. Wang et al. (Aging Cell, 2025, ref. 29), already cited in our manuscript, demonstrated through WGCNA that *Spp1* co-segregates with established inner cortex markers (*Akr1c18*, *Cyp2f2*, *Pik3c2g*, *Dhcr24*) in a sex- and age-dependent co-expression module — providing independent computational support consistent with our spatial findings. Furthermore, Dolfi et al. (Cell Reports, 2022) demonstrated that adrenal gland macrophages show pronounced sex-dimorphic distribution that is directly dependent on the presence of the X-zone, providing biological context for the SPP1-expressing macrophage population enriched in females in our dataset. Together, these independent lines of evidence provide orthogonal support for our spatial observations without requiring additional wet-lab experiments.

We have framed SPP1-integrin signalling explicitly as a priority target for future functional investigation, including the use of *Spp1* knockout mice, which are viable and fertile and would represent an ideal model to test whether loss of osteopontin affects X-zone morphology or involution dynamics. We also note that Reviewer #2, having carefully evaluated the revised manuscript, acknowledged the SPP1-related observations and considered additional functional experiments to be beyond the scope of the current study, a judgment we respectfully share.

The identification and correction of a substantial technical bias, a 3.65-fold sequencing depth imbalance between male and female libraries (25.0 GB vs. 6.85 GB), and the subsequent removal of conclusions that did not survive more rigorous analytical treatment, reflects a commitment to scientific rigor rather than evidence against it. Retaining conclusions known to be influenced by technical artefacts would have constituted the greater breach of rigor. We have been fully transparent about this reanalysis in the revised Methods and Limitations sections. The core findings of the study, the molecular identity of the X-zone, the spatial restriction of sexual dimorphism to inner cortical zones, and the sex-specific intercellular communication architecture, are robust across the revised analytical framework and consistent with multiple independent published datasets.

Next, we have removed the CYP2F1 staining panel and all interpretations presenting CYP2F1 as a protein-level surrogate for CYP2F2. The immunohistochemical validation panel has been revised to present four markers: AEBP1 and HMOX1 (male-biased), and NREP and AKR1D1 (female-biased/X-zone-enriched). In the revised manuscript, *Cyp2f2* is discussed exclusively at the transcriptomic level and contextualized through the published IHC validation by Lyu et al. (Endocrinology, 2020, ref. 55).

We have re-examined the original spatial expression plots, confirmed the duplication, and replaced the affected panels in Figure 3 with updated spatial maps generated from the revised dataset. The figure legends have been corrected accordingly.

Reviewer #4 (Remarks to the Author):

I co-reviewed this manuscript with one of the reviewers who provided the listed

reports. This is part of the Communications Biology initiative to facilitate training in peer review and to provide appropriate recognition for Early Career Researchers who co-review manuscripts.

Response to Reviewer #4

We thank Reviewer #4 for participating in the peer review process of this manuscript and for their contribution to the evaluation alongside the primary reviewer. We appreciate the Communications Biology initiative to support Early Career Researchers in developing peer review expertise, and we hope that our detailed responses to the reviewers' comments and the revised manuscript will meet their expectations.
