# Supplementary material for: Single-cell spatial transcriptomics reveals sex-dependent gene expression and intercellular signalling in mouse adrenal cortex
Source: Commun Biol. 2026 Jul 14;9:1480. doi: 10.1038/s42003-026-10697-9 (PMC13740276; doi:10.1038/s42003-026-10697-9)
Supplement: Supplementary file 2 [file 42003_2026_10697_MOESM2_ESM.pdf]

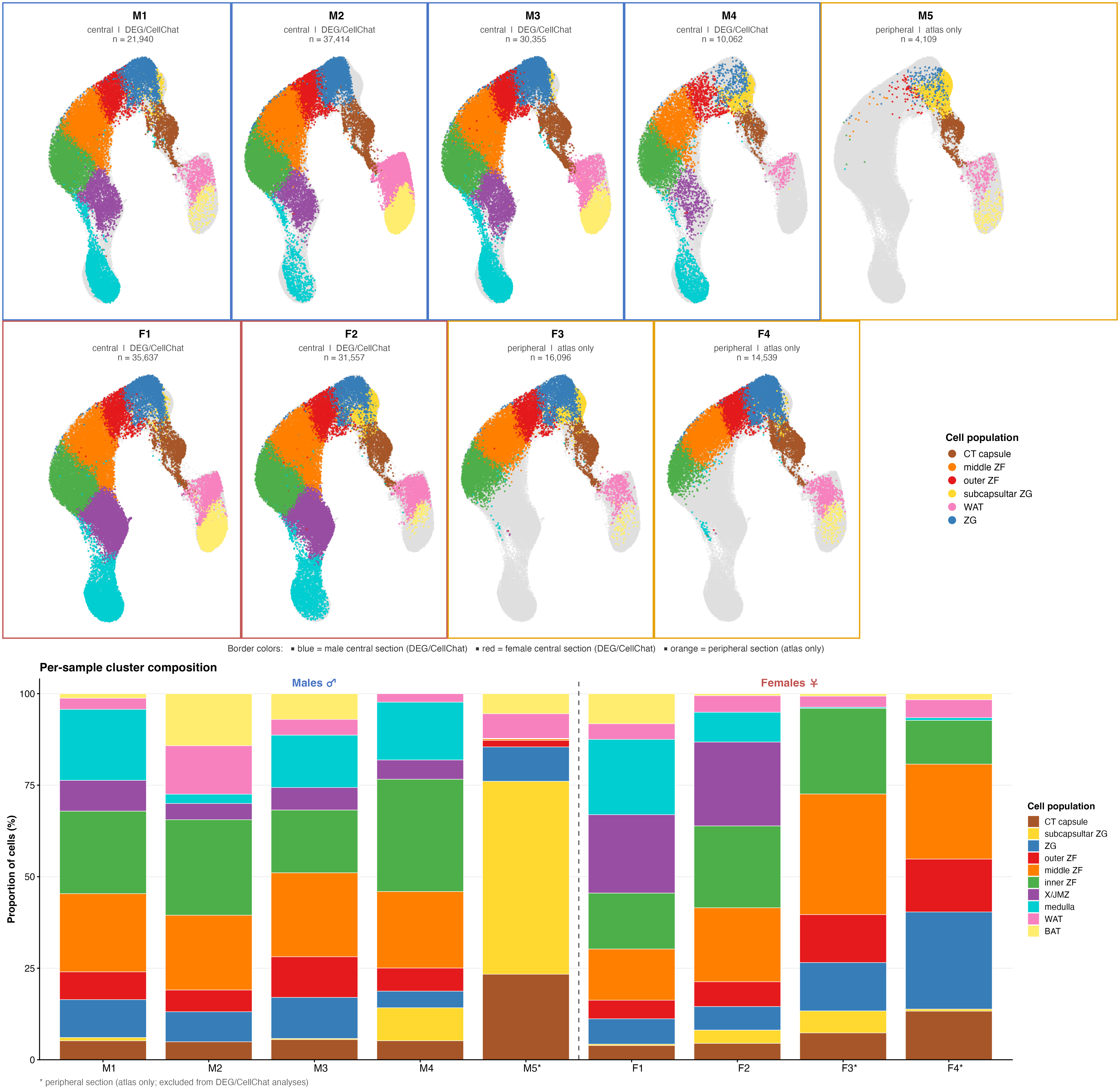
M1
central | DEG/CellChat
n = 21,940
M2
central | DEG/CellChat
n = 37,414
M3
central | DEG/CellChat
n = 30,355
M4
central | DEG/CellChat
n = 10,062
M5
peripheral | atlas only
n = 4,109
F1
central | DEG/CellChat
n = 35,637
F2
central | DEG/CellChat
n = 31,557
F3
peripheral | atlas only
n = 16,096
F4
peripheral | atlas only
n = 14,539
Cell population
CT capsule
middle ZF
outer ZF
subcapsultar ZG
WAT
ZG
Border colors: ▪ blue = male central section (DEG/CellChat) ▪ red = female central section (DEG/CellChat) ▪ orange = peripheral section (atlas only)
Per-sample cluster composition
Males ♂
Females ♀
Proportion of cells (%)
0
25
50
75
100
M1
M2
M3
M4
M5*
F1
F2
F3*
F4*
Cell population
CT capsule
subcapsultar ZG
ZG
outer ZF
middle ZF
inner ZF
X/JMZ
medulla
WAT
BAT
* peripheral section (atlas only; excluded from DEG/CellChat analyses)

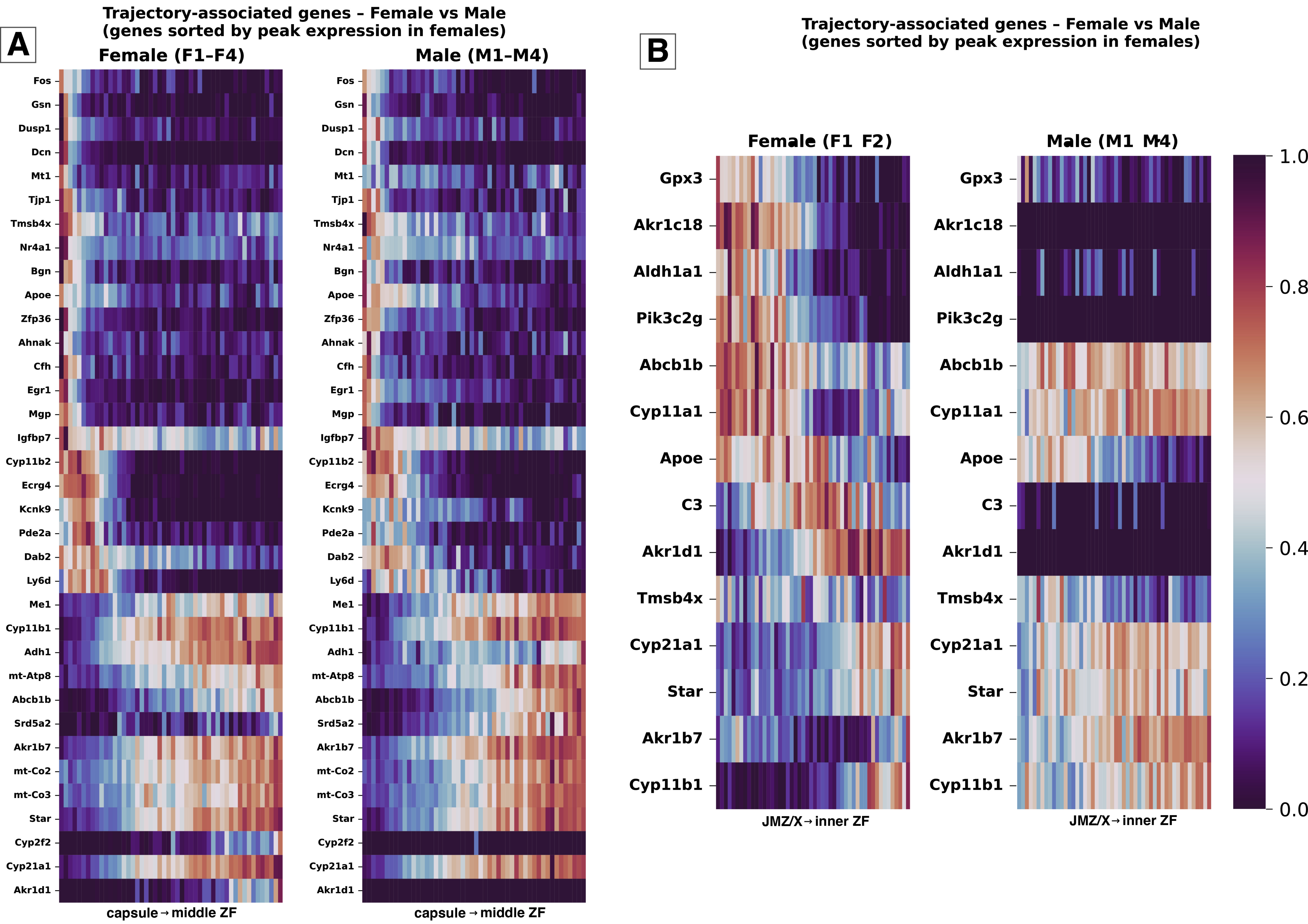

A
Trajectory-associated genes – Female vs Male
(genes sorted by peak expression in females)
Female (F1–F4)
Male (M1–M4)
Fos
Gsn
Dusp1
Dcn
Mt1
Tjp1
Tmsb4x
Nr4a1
Bgn
Apoe
Zfp36
Ahnak
Cfh
Egr1
Mgp
Igfbp7
Cyp11b2
Ecrg4
Kcnk9
Pde2a
Dab2
Ly6d
Me1
Cyp11b1
Adh1
mt-Atp8
Abcb1b
Srd5a2
Akr1b7
mt-Co2
mt-Co3
Star
Cyp2f2
Cyp21a1
Akr1d1
capsule → middle ZF
capsule → middle ZF
B
Trajectory-associated genes – Female vs Male
(genes sorted by peak expression in females)
Female (F1 F2)
Male (M1 M4)
Gpx3
Akr1c18
Aldh1a1
Pik3c2g
Abcb1b
Cyp11a1
Apoe
C3
Akr1d1
Tmsb4x
Cyp21a1
Star
Akr1b7
Cyp11b1
JMZ/X → inner ZF
JMZ/X → inner ZF
1.0
0.8
0.6
0.4
0.2
0.0

Supplementary Figure S1. Reproducibility of cell population assignments across individual tissue sections. (A) UMAP plots showing Harmony-integrated embeddings for each of the nine individual tissue sections (five male, four female), with cells colored by assigned cluster identity. Border color indicates section type: blue = male central section included in pseudobulk DEG and CellChat analyses; red = female central section included in pseudobulk DEG and CellChat analyses; orange = peripheral section used for atlas construction only. Cell numbers per section are indicated. (B) Stacked bar plots showing the proportional composition of cell populations in each individual section.

Supplementary Figure S2. Cross-sex comparison of trajectory-associated gene expression. (A) Trajectory-associated genes along the capsule-to-middle ZF axis shown for female (F1–F4, left) and male (M1–M4, right) samples. Genes consistently identified in ≥2 female samples are ordered by peak pseudotime position in females. Colour intensity indicates min-max normalized mean expression per pseudotime bin. (B) Trajectory-associated genes along the JMZ/X-to-inner ZF axis shown for female (F1, F2, left) and male (M1–M4, right) samples. Genes consistently identified in ≥2 female samples are ordered by peak pseudotime position in females. Colour intensity indicates min-max normalized mean expression per pseudotime bin.
